# Self-(in)compatibility in apricot germplasm is controlled by two major loci, *S* and *M*

**DOI:** 10.1186/s12870-017-1027-1

**Published:** 2017-04-26

**Authors:** Juan Vicente Muñoz-Sanz, Elena Zuriaga, Inmaculada López, María L. Badenes, Carlos Romero

**Affiliations:** 1 Fruit Tree Breeding Department, Instituto Valenciano de Investigaciones Agrarias (IVIA), CV-315, Km. 10,7., 46113 Moncada, Valencia Spain; 2 Department of Biochemistry, University of Missouri, 117 Schweitzer Hall, 65211 Columbia , MO USA; 3 Instituto de Biología Molecular y Celular de Plantas (IBMCP), Universidad Politécnica de Valencia-Consejo Superior de Investigaciones Científicas, Camino de Vera, 46022 Valencia, Spain

**Keywords:** Apricot, *Prunus*, Self-(in)compatibility, *S*-locus, *S*-alleles, Modifiers, *M*-locus

## Abstract

**Background:**

Apricot (*Prunus armeniaca* L.) exhibits a gametophytic self-incompatibility (GSI) system and it is mostly considered as a self-incompatible species though numerous self-compatible exceptions occur. These are mainly linked to the mutated *S*_C_-haplotype carrying an insertion in the *S*-locus *F-box* gene that leads to a truncated protein. However, two *S*-locus unlinked pollen-part mutations (PPMs) termed *m* and *m’* have also been reported to confer self-compatibility (SC) in the apricot cultivars ‘Canino’ and ‘Katy’, respectively. This work was aimed to explore whether other additional mutations might explain SC in apricot as well.

**Results:**

A set of 67 cultivars/accessions with different geographic origins were analyzed by PCR-screening of the *S*- and *M*-loci genotypes, contrasting results with the available phenotype data. Up to 20 *S*-alleles, including 3 new ones, were detected and sequence analysis revealed interesting synonymies and homonymies in particular with *S*-alleles found in Chinese cultivars. Haplotype analysis performed by genotyping and determining linkage-phases of 7 SSR markers, showed that the *m* and *m’* PPMs are linked to the same *m*_0−_haplotype. Results indicate that *m*_0_-haplotype is tightly associated with SC in apricot germplasm being quite frequent in Europe and North-America. However, its prevalence is lower than that for *S*_C_ in terms of frequency and geographic distribution. Structures of 34 additional *M*-haplotypes were inferred and analyzed to depict phylogenetic relationships and *M*_1–2_ was found to be the closest haplotype to *m*_0._ Genotyping results showed that four cultivars classified as self-compatible do not have neither the *S*_C_- nor the *m*_0_-haplotype.

**Conclusions:**

According to apricot germplasm *S*-genotyping, a loss of genetic diversity affecting the *S*-locus has been produced probably due to crop dissemination. Genotyping and phenotyping data support that self-(in)compatibility in apricot relies mainly on the *S*- but also on the *M*-locus. Regarding this latter, we have shown that the *m*_0_-haplotype associated with SC is shared by ‘Canino’, ‘Katy’ and many other cultivars. Its origin is still unknown but phylogenetic analysis supports that *m*_0_ arose later in time than *S*_C_ from a widely distributed *M*-haplotype. Lastly, other mutants putatively carrying new mutations conferring SC have also been identified deserving future research.

**Electronic supplementary material:**

The online version of this article (doi:10.1186/s12870-017-1027-1) contains supplementary material, which is available to authorized users.

## Background

Gametophytic self-incompatibility (GSI) is a system widely distributed in the plant kingdom [1] that prevents self-fertilization favoring outcrossing [2]. GSI specific recognition is under the control of a multi-allelic locus, termed *S*-locus, containing at least two linked genes: a pistil expressed S-RNase [3] and the pollen expressed *S*-locus *F-box* [4–6]. *S*-locus F-box proteins are thought to be components of E3 ubiquitin ligase complexes that recognize non-self *S*-RNases promoting their ubiquitination and degradation by the 26S proteasome proteolytic pathway [7, 8]. Recently, the collaborative model proposed in Solanaceae (*Petunia*) suggests that several F-box proteins are necessary to recruit *S*-RNases for degradation [9, 10]. This system seems to be extended to other plant families exhibiting GSI such as Rosaceae and particularly the Maloideae subfamily [10, 11]. However, *Prunus* does not seem to follow this model since knock-out of the *S*-locus F-box (*SFB*) gene leads to self-compatibility (SC) in contrast with the observations in Solanaceae. Reasons behind this behavior have been speculated for a long time [12, 13] but only recently evidence have been provided supporting *S*-locus F-box like-2 protein as a ‘general inhibitor’ that detoxifies S-RNases unspecifically, unless affected by SFB [14].

In general, self-incompatibility (SI) trait predominates in stone fruits (*Prunus* genus) in accordance with the high degree of heterozygosity showed by many of these species. However, different ‘degrees’ of SC have been detected in this genus ranging from sweet cherry, almost strictly self-incompatible (with a few exceptions), or apricot, mainly self-incompatible (SC is restricted to European cultivars), to complete SC in peach [15]. SC is mostly related to mutations in the *S*-locus genes and mutations affecting both *S*-*RNases* and *SFBs* have been detected in many *Prunus* species [13, 16]. In apricot (*Prunus armeniaca* L.) the *S*_C_-allele known to confer SC has been well characterized showing that a 358 bp insertion in the *SFB* gene leads to a putative truncated protein lacking the two essential 3′-hypervariable domains HVa and HVb [17]. Furthermore, the origin and dissemination of *S*_C_ has also been studied, identifying the non-mutated ancestor *S*_8_-allele and detecting its presence in accessions from different geographic areas [18]. In fact, most *S*-genotyped self-compatible apricot cultivars have been shown to carry the *S*_C_-allele [18–22].

Along with the *S*-locus specific products, other *S*-locus unlinked factors are also necessary for the GSI system to work. These factors known as ‘modifiers’ were firstly identified in Solanaceae [23]. Nevertheless, genetic evidence supporting modifiers have also been accumulated in other species including *Prunus spp*. [17, 24]. In apricot, pollen-part mutations (PPMs) conferring SC by putatively affecting modifiers have been identified in the Spanish local cultivar ‘Canino’ and the North-American one ‘Katy’. Both PPMs were mapped at the distal end of chr.3 within the so-called *M*- and *M*’-loci, respectively [25, 26]. Given their distinct geographic origins and the significant genetic distance estimated between both cultivars, it was initially hypothesized that these two PPMs could have arisen independently but affecting the same gene [26].

In this context, we thought that additional *S*-locus unlinked mutations (and not just restricted to the *M*-locus) might also be present in apricot germplasm. Thus, in this study we have genotyped both the *S*- and *M*-loci in a wide set of apricot cultivars and accessions from different geographic origins, by using *SFB* and *S*-*RNase* introns and *M*-locus linked SSR markers. These data were combined with those of phenotyping to dissect SC causes and distribution in apricot germplasm.

## Results

### Self-incompatibility versus self-compatibility in apricot

A set of 67 apricot cultivars and accessions (hereinafter referred only as accessions) representing a wide range of geographic origins has been analyzed. Information about pedigree was only available for a few of them but it was quite useful to support genotyping data. Blooming time and self-(in)compatibility phenotypes were scored according to literature reports. Thus, apricot accessions were classified into 5 gross classes (early, mid-early, mid, mid-late and late) regarding the first phenotype and as self-incompatible or self-compatible regarding the second (Table 1).

**Table 1:** Country of origin, pedigree, self-(in)compatibility and blooming time phenotypes of the analyzed apricot accessions

	Cultivar	Source^b^	Geog. area^c^	Country of origin^d^	Pedigree^e^	SI/SC^e^	Blooming time^e^
1	Alba	CAPA	WE	Italy	Unknown	?	Mid^f^
2	ASP^a^	FM	WE	Spain (V)	Unknown (isolated tree) [25]	?	Mid^f^
3	Aurora	CAPA	NA	USA	RR17–62 × NJA-13 [26]	SI [73]	Mid [74]
4	Bebecou	MAGRAMA	SE/NAf	Greece	Unknown [41]	SC [73]	Mid-early [41]
5	Bergeron	MAGRAMA	WE	France	Unknown [41]	SC [73]	Mid-late [41, 47]
6	Budapest	St. Istvan U	EE	Hungary	‘Nancy’ × (‘Acme’, ‘Hungarian Best’, ‘Kései Rózsa’) [16]	SC [16]	Mid-late [75]
7	Búlida	MAGRAMA	SE/NAf	Spain (M)	Unknown [41]	SC [73]	Mid [41]
8	Canino	IVIA	WE	Spain (V)	Unknown [41]	SC [73]	Mid [41]
9	Canino 9–7	IVIA	WE	Spain (V)	Clonal selection from Canino [27]	?	Mid [27]
10	Canino 14–4	IVIA	WE	Spain (V)	Clonal selection from Canino [27]	?	Mid [27]
11	Canino 14–6	IVIA	WE	Spain (V)	Clonal selection from Canino [27]	?	Mid [27]
12	Castlebrite	CEBAS	NA	USA	o.p. (Perfection × Castleton) [76]	SC [73]	Mid-late^f^
13	Castleton	CEBAS	NA	USA	Perfection × Newcastle [29]	SC [76]	Mid [41]
14	Ceglédi óriás	St. Istvan U	EE	Hungary	Unknown (local selection) [16]	SI [32]	Mid [35]
15	Colorao	CEBAS	SE/NAf	Spain (M)	Unknown [41]	↗ sterile [73]	Mid [41]
16	Corbató	MAGRAMA	WE	Spain (V)	Unknown [25]	?	Mid-late [74]
17	Cow-1	CAPA	WE	France	INRA	?	Mid [35]
18	Cow-2	CAPA	WE	France	INRA	?	?
19	Cristalí	FM	WE	Spain (V)	Unknown [25]	?	Mid [74]
20	Currot	IVIA	WE	Spain (V)	Unknown [25]	SC [73]	Early [74]
21	Dulcinea	CAPA	WE	Italy	Unknown (Toscana variety)	SC [73]	Mid [35]
22	Effect	St. Istvan U	EE	Ukraine	Krupnolodnyi o.p. [16]	SC [16]	Late [77]
23	Ezzine	CAPA	SE/NAf	Tunisia	INRAT	?	Early^f^
24	Fergani	St. Istvan U	EE	Former USSR	Unknown	?	?
25	Galta Roja^a^	CAPA	WE	Spain (V)	Unknown [25]	SC [73]	Mid-early [41]
26	GVV	FM	WE	Spain (V)	Unknown (isolated tree) [25]	?	?
27	Gandía	FM	WE	Spain (V)	Unknown [25]	?	Mid-early [74]
28	Gavatxet	FM	WE	Spain (V)	Unknown [25]	?	Mid [74]
29	Ginesta	IVIA	WE	Spain (V)	Unknown [25]	SC^e^	Mid-early [74]
30	Goldrich	IVIA	NA	USA	Sunglo × Perfection [76]	SI [73]	Mid [41, 47]
31	Gönci Magyar	St. Istvan U	EE	Hungary	Clone/hybrid? of Hungarian Best [16]	SC [16]	Mid-late [75]
32	Harcot^a^	IVIA	NA	Canada	[(Geneva × Naramata) × Morden 604] × NJA1(Phelps × Perfection) [76]	SI [73]	Mid [41]
33	Hargrand	St. Istvan U	NA	Canada	V51092 [(Reliable × o.p.) × o.p.] × NJA1 (Phelps × Perfection) [76]	SI [73]	Late^f^
34	Harlayne	IVIA	NA	Canada	V51092 [(Reliable × o.p.) × o.p.] × Sunglo [76]	SC [73]	Mid-late [41, 77]
35	Henderson	IVIA	NA	USA	Unknown [76]	SC?	Mid-late [41, 77]
36	Katy	IVIA	NA	USA	Zaiger’s genetics (USA) [35]	SC [35]	Early^f^
37	Kech-pshar	St. Istvan U	EE	Uzbekistan	Unknown (local selection) [16]	?	?
38	K. Pozdnii^a^	St. Istvan U	EE	Ukraine	Unknown (chance seedling) [16]	SC [16]	Mid-late [75]
39	Lambertin-1	CEBAS	NA	USA	[Perfection × (Royal x Blush)] o.p. × (Perfection × Royal) [35]	SI [73]	Mid [35]
40	Lito	IVIA	SE/NAf	Greece	SEO × Tyrinthos [32]	SC [32]	Mid-late^f^
41	Manrí	FM	WE	Spain (V)	Unknown [25]	?	Mid-early [74]
42	Mari de Cenad	St. Istvan U	EE	Romania	Unknown [16]	SC [16]	?
43	Mariem	CAPA	SE/NAf	Tunisia	2nd generation (Bergeron × Ouardi) × (Carraut × Crossa-Raynaud) [35]	?	Mid [35]
44	Martinet	FM	WE	Spain (V)	Unknown [25]	?	Mid [74]
45	Mitger	IVIA	WE	Spain (V)	Unknown [25]	SC^e^	Mid [35]
46	Moniquí	CEBAS	SE/NAf	Spain (M)	Unknown [41]	SI [73]	Mid [41]
47	Ninfa	CAPA	WE	Italy	Ouardi × Tyrinthos [35]	SC [73]	Mid-early [47]
48	Orange Red	IVIA	NA	USA	Lasgerdi Mashhad × NJA2 [35]	SI [73]	Mid [41, 47]
49	Ouardi^a^	IVIA	SE/NAf	Tunisia	Canino × Hamidi [41]	SI [73]	Mid-early [41]
50	Palabras	IVIA	WE	Spain (V)	Unknown [25]	SC^e^	Mid-early [74]
51	Palau	IVIA	WE	Spain (V)	Unknown [25]	SC^e^	Mid-early [74]
52	Patterson	CEBAS	NA	USA	F_2_ seedling Perfection × unknown [76]	SC [73]	Mid-late [76]
53	Perla	CAPA	WE	Italy	ANFIC (Italy)	?	Mid-early [35]
54	Portici^a^	FM	WE	Italy	Unknown [41]	SC [73]	Mid-early [41]
55	Rojo de Carlet	IVIA	WE	Spain (V)	Unknown [25]	SC^e^	Mid [74]
56	Rózsakajszi	St. Istvan U	EE	Hungary	Local selection Nagykórös [16]	SC [16]	Mid-late [75]
57	Sayeb^a^	CEBAS	SE/NAf	Tunisia	Canino × Hamidi [41]	SC [73]	Mid-early [41]
58	SEO^a^	IVIA	NA	USA	Unknown [76]	SI [73]	Mid-late [41]
59	Shalah^a^	St. Istvan U	EE	Armenia	Unknown	SC/SI? [73]	Late [77]
60	Stella	IVIA	NA	USA	Unknown [41]	SI [73]	Late [41, 77]
61	S. Mammut^a^	St. Istvan U	EE	Hungary	Unknown (local selection)	SI [73]	Mid [75]
62	Tadeo	IVIA	WE	Spain (V)	Unknown [25]	SC [73]	Mid-late [74]
63	Tirynthos	IVIA	SE/NAf	Greece	Unknown [41]	SC [73]	Early [41]
64	Trevatt	CEBAS	NA	Australia	Unknown [35]	SC [32]	Mid-late [41]
65	Veecot	IVIA	NA	Canada	o.p. from Reliable [76]	SI [73]	Mid [41]
66	Velázquez	MAGRAMA	SE/NAf	Spain (M)	Unknown	SI [73]	Late^f^
67	Xirivello	CAPA	WE	Spain (V)	Unknown [25]	?	Mid-late [74]

^a^Synonyms and acronyms: ASP ‘*Albaricoquero Sin Polen’*; Galta Roja ‘Galta Roja de Mitger’; GVV ‘Galta Vermella Valenciana’; Ouardi ‘Priana’; Portici ‘Portici-6’; Sayeb ‘Beliana’; SEO ‘Stark Early Orange’ and Shalah ‘Erevani’; K. Pozdnii ‘Konservnyi Pozdnii’; S. Mammut ‘Szegedi Mammut’

^b^Material sources (see Methods)

^c^Main geographic areas defined in this study: Western Europe (WE); Southern Europe/North Africa (SE/NAf); Eastern Europe (EE) and North America (NA)

^d^Accessions from Spain come from two different regions: Valencia (V) and Murcia (M). The first ones were assigned to WE and the second ones to SE/NAf

^e^References for pedigree are indicated when available. Reports for SI/SC and blooming time phenotypes are also indicated marking with (?) when no consistent data were found: García et al. [41]; Halázs et al. [27]; Della Strada et al. [76]; Halázs et al. [18]; Orero et al. [35]; Brooks and Olmo [32]; Russell [73]; Syrgianidis and Mainou [74]; Burgos et al. [47]; Badenes et al. [75]; Massai [77]; Nyujtó et al. [78]; Mehlenbacher et al. [36]

^f^IVIA’s own data (see footnotes in Table 3)

Self-(in)compatibility phenotype data were completed with our own results when adult trees were available. In this regard, self-pollination tests were used to determine (8) or to confirm (13) self-(in)compatibility phenotypes but also to obtain progenies useful to search for *S*-locus unlinked mutations. Data suggest that 5 accessions are self-incompatible (‘Aurora’, ‘Cow-2’, ‘Perla’, ‘Veecot’ and ‘Velázquez’) while the remaining 16 show variable fruit-setting ranging from 0.5% (‘Búlida’) to 55% (‘Ninfa’), being recorded as self-compatibles (Table 2). Some progeny could be finally obtained from all accessions of this latter group except from ‘Búlida’. Those accessions with a progeny large enough were subsequently analyzed for non-*S*-locus mutations by *S*-genotyping embryos. The self-compatible controls (‘Canino’ and ‘Katy’), already studied, and the *S*_C_ homozygote cultivar ‘Galta Roja’ were excluded. In summary, 40 out of the 67 accessions analyzed were classified as self-compatible, 16 as self-incompatible, 2 as male-sterile and the 9 remaining as undetermined (Table 3).

**Table 2:** Number of bagged flowers, fruit-setting percentage and SI/SC deduced phenotype in self-pollination tests

Cultivar/Accession	Bagged flowers	Fruit-Setting	% Setting	Phenotype	Progeny^d^
Alba^a^	355	37	10.4	SC	1
Aurora	350	0	0	SI	--
Bebecou	760	108	14.2	SC	96
Búlida	200	1	0.5	SC?^b^	--
Canino	412	99	24.0	SC	99
Castlebrite	300	36	12.0	SC	2
Corbató^a^	320	52	16.3	SC	44
Cow-1^a^	200	8	4.0	SC	7
Cow-2^a^	315	0	0	SI	--
Cristalí^a^	200	24	12.0	SC	13
Dulcinea	850	111	13.1	SC	104
Ezzine^a^	350	160	45.7	SC	21
Galta Roja	775	106	13.7	SC	106
Katy	731	80	10.9	SC	80
Mariem^a^	450	11	2.4	SC?^c^	11
Ninfa	400	221	55.3	SC	12
Perla^a^	370	0	0	SI	--
Portici	850	63	7.4	SC	59
Tadeo	375	14	3.7	SC	5
Veecot	450	0	0	SI	--
Velázquez	270	0	0	SI	--

^a^Cultivars and accessions of unknown SI/SC phenotype

^b^In general, fruit-setting percentages below 2–3% should not be undoubtedly associated to SC since some degree of pollen contamination cannot be fully discarded

^c^ *S*-genotyping of the progeny suggested some degree of non-self pollination and therefore SC could not be confirmed

^d^Number of individual plants obtained from the set fruits

**Table 3:** *S*- and *M*-haplotypes assigned to the apricot accessions analyzed in this study

	Cultivar	SI/SC	*S*-genot^d^	*M*-genot^e^		Cultivar	SI/SC	*S*-genot	*M*-genot
1	Alba	SC^b^	*S*_1_/***S***_**C**_	*M*_1–1_/*M*_3_	35	Henderson	SC?	*S*_3_/*S*_17_	*M*_2–0_/*M*_16_
2	ASP	↗sterile^b,c^	*S*_5_/***S***_**C**_	*M*_4–0_/*M*_5–1_	36	Katy^a^	SC/SC^b^	*S*_1_/*S*_2_	*M*_3_/***m***_**0–0**_
3	Aurora^a^	SI	*S*_1_/*S*_17_	*M*_3_/*M*_11_	37	Kech-pshar^a^	?	*S*_15_/*S*_Z_	*M*_?_/*M*_?_
4	Bebecou	SC	*S*_6_/***S***_**C**_	*M*_4–0_/*M*_4–2_	38	Konservnyi P. ^a^	SC	*S*_2_/***S***_**C**_	*M*_4–1_/*M*_8–1_
5	Bergeron^a^	SC	*S*_2_/***S***_**C**_	*M*_4–0_/*M*_8–0_	39	Lambertin-1^a^	SI	*S*_1_/*S*_2_	*M*_1–0_/*M*_17_
6	Budapest^a^	SC	*S*_2_/***S***_**C**_	*M*_1–1_/*M*_12_	40	Lito	SC^b^	*S*_6_ **/** ***S***_**C**_	*M*_6_/*M*_10_
7	Búlida	SC	*S*_5_/***S***_**C**_	*M*_4–1_/*M*_5–1_	41	Manrí	?	***S***_**C**_ **/** ***S***_**C**_	***m***_**0–0**_ **/** ***m***_**0–0**_
8	Canino^a^	SC/SC^b^	*S*_2_/***S***_**C**_	*M*_1–0_/***m***_**0–0**_	42	Mari de Cenad^a^	SC	***S***_**C**_/*S*_19_	*M*_8–0_/*M*_18_
9	Canino 9–7	?	*S*_2_/***S***_**C**_	*M*_1–0_/***m***_**0–0**_	43	Mariem	SC?^b^	*S*_7_/*S*_20_	*M*_1–0_/*M*_8–2_
10	Canino 14–4	SC^b^	*S*_2_/***S***_**C**_	*M*_1–0_/***m***_**0–0**_	44	Martinet	?	*S*_2_/*S*_2_	***m***_**0–0**_ **/** ***m***_**0–1**_
11	Canino 14–6	SC^b^	*S*_2_/***S***_**C**_	*M*_1–0_/***m***_**0–0**_	45	Mitger	SC^b^	***S***_**C**_ **/** ***S***_**C**_	*M*_5–0_/*M*_5–0_
12	Castlebrite	SC	*S*_2_/*S*_2_	*M*_3_/***m***_**0–0**_	46	Moniquí^a^	SI	*S*_2_/*S*_6_	*M*_4–1_/*M*_14–0_
13	Castleton	SC	*S*_1_/*S*_2_	*M*_3_/***m***_**0–0**_	47	Ninfa	SC	*S*_7_/***S***_**C**_	*M*_7–0_/*M*_10_
14	Ceglédi óriás^a^	SI	*S*_8_/*S*_9_	*M*_12_/*M*_13_	48	Orange Red	SI	*S*_6_/*S*_17_	*M*_2–0_/*M*_2–1_
15	Colorao^a^	↗sterile	*S*_5_/***S***_**C**_	*M*_4–0_/*M*_4–0_	49	Ouardi^a^	SI	*S*_2_/*S*_7_	*M*_1–0_/*M*_7–0_
16	Corbató	SC^b^	*S*_2_/*S*_5_	*M*_5–0_/***m***_**0–0**_	50	Palabras	SC/SC^b^	***S***_**C**_ **/** ***S***_**C**_	***m***_**0–0**_ **/** ***m***_**0–0**_
17	Cow-1	SC^b^	*S*_1_/*S*_31_	*M*_3_/***m***_**0–0**_	51	Palau^a^	SC/SC^b^	***S***_**C**_ **/** ***S***_**C**_	***m***_**0–0**_ **/** ***m***_**0–0**_
18	Cow-2	SI^b^	*S*_20_/*S*_31_	*M*_1–0_/***m***_**0–0**_	52	Patterson	SC	*S*_1_/***S***_**C**_	*M*_1–3_/*M*_3_
19	Cristalí	SC^b^	*S*_20_/***S***_**C**_	*M*_5–0_/***m***_**0–0**_	53	Perla	SI^b^	*S*_2_/*S*_20_	*M*_1–2_/*M*_15–1_
20	Currot^a^	SC/SC^b^	***S***_**C**_ **/** ***S***_**C**_	***m***_**0–0**_ **/** ***m***_**0–0**_	54	Portici	SC/SC^b^	*S*_2_/*S*_20_	*M*_1–4_/***m***_**0–0**_
21	Dulcinea	SC	*S*_2_/***S***_**C**_	*M*_7–3_/*M*_14–1_	55	Rojo Carlet	SC^b^	***S***_**C**_ **/** ***S***_**C**_	*M*_4–0_/*M*_5–0_
22	Effect^a^	SC	*S*_8_/***S***_**C**_	*M*_8–0_/*M*_12_	56	Rózsakajszi^a,f^	SC	*S*_2_/***S***_**C**_	*M*_1–0_/*M*_12_
23	Ezzine	SC^b^	*S*_24_/***S***_**C**_	*M*_1–0_/*M*_7–1_	57	Sayeb^a^	SC	*S*_7_/***S***_**C**_	*M*_1–0_/*M*_7–2_
24	Fergani	?	*S*_V_/*S*_X_	*M*_?_/*M*_?_	58	Shalah^a^	SC	*S*_*5*_/*S*_11_	*M*_8–1_/*M*_19_
25	GaltaRoja	SC/SC^b^	***S***_**C**_ **/** ***S***_**C**_	*M*_4–0_/*M*_5–2_	59	SEO	SI	*S*_6_/*S*_17_	*M*_2–0_/*M*_6_
26	GVV	?	*S*_2_/***S***_**C**_	*M*_4–0_/*M*_5–2_	60	Stella	SI	*S*_6_/*S*_20_	*M*_9_/*M*_9_
27	Gandía	?	***S***_**C**_ **/** ***S***_**C**_	***m***_**0–0**_ **/** ***m***_**0–0**_	61	Szegedi M.	SI	*S*_8_/*S*_9_	*M*_12_/*M*_13_
28	Gavatxet	?	*S*_20_/***S***_**C**_	***m***_**0–0**_ **/** ***m***_**0–0**_	62	Tadeo	SC	*S*_20_/***S***_**C**_	*M*_15–0_/***m***_**0–0**_
29	Ginesta^a^	SC/SC^b^	***S***_**C**_ **/** ***S***_**C**_	***m***_**0–0**_ **/** ***m***_**0–0**_	63	Tirynthos	SC	***S***_**C**_ **/** ***S***_**C**_	*M*_10_/*M*_10_
30	Goldrich^a^	SI/SI^b^	*S*_1_/*S*_2_	*M*_1–0_/*M*_2–0_	64	Trevatt	SC	*S*_2_/***S***_**C**_	*M*_1–0_/***m***_**0–0**_
31	Gönci Magyar^a^	SC	*S*_8_/***S***_**C**_	*M*_8–0_/*M*_12_	65	Veecot	SI/SI^b^	*S*_2_/*S*_20_	*M*_2–0_/*M*_3_
32	Harcot^a^	SI/SI^b^	*S*_1_/*S*_4_	*M*_1–0_/*M*_2–2_	66	Velázquez	SI	*S*_5_/*S*_20_	*M*_4–0_/*M*_4–1_
33	Hargrand^a^	SI	*S*_1_/*S*_2_	*M*_1–0_/*M*_2–0_	67	Xirivello	?	***S***_**C**_ **/** ***S***_**C**_	*M*_4–0_/*M*_4–1_
34	Harlayne	SC	*S*_3_/*S*_20_	*M*_2–0_/*M*_9_					

^a^Cultivars previously *S*-genotyped by Halázs et al. [18]; Vilanova et al. [19]; Halázs et al. [20]; Zuriaga et al. [26]; Halázs et al. [27]; Burgos et al. [42]; Alburquerque et al. [79]

^b^Own data on SI/SC phenotype obtained in this work (see Table 2). Additionally, SC had been observed in a set of accessions grown under insect-proof screen house at IVIA (‘Rojo de Carlet’, ‘Mitger’, ‘Palabras’, ‘Palau’, ‘Currot’, ‘Ginesta’, ‘Canino 14–4’ and ‘Canino 14–6’) showing moderate fruit-setting (not quantified) across several years

^c^Male-sterility in the ASP accession was indicated by shrunken pale anthers

^d^ *S*-allele nomenclature is proposed according to Vilanova et al. [19]; Halázs et al. [27]; Zhang et al. [29]; Wu et al. [30]; Gu et al. [31] and Halázs et al. [28]. *S*-haplotype associated with SC (*S*_C_) is written in bold

^e^ *M*-haplotypes were named with two digits. The first one corresponds to the *M*-haplotype ‘main class’ and the second to the subtype. *M*-haplotype variants associated with SC (*m*_0–0_ and *m*_0–1_) are written in bold. Haplotypes designated by *M*_?_ could not be defined

^f^ *S*-genotype determined for Rózsakajszi (*S*_2_ *S*_C_) was not in agreement with that previously reported by Halázs et al. [18] (*S*_C_ *S*_C_). Reasons for this discrepancy are still unknown

### *S*-genotyping: identification of new *S*-alleles

All the 67 accessions were *S*-genotyped using primer pairs amplifying different fragments of the *S*-haplotype region: the first and second *S*-*RNase* introns as well as the 5′-UTR *SFB* intron (Table 3). *S*-alleles were determined by comparing intron size patterns with previously *S*-genotyped accessions and, when needed, sequence analyses supported these assignments. Fragment analyses of the PCR products obtained with 5 primer combinations allowed us to detect up to 20 different *S*-alleles. Seventeen out of them had already been reported in apricot (*S*_1_, *S*_2_, *S*_3_, *S*_4_, *S*_5_, *S*_6_, *S*_7_, *S*_8_, *S*_9_, *S*_11_, *S*_15_, *S*_17_, *S*_18_, *S*_19_, *S*_20_, *S*_24_ and *S*_C_) and in the present work were designed basically according to the nomenclature established by Vilanova et al. [19], Halázs et al. [27] and Halázs [28], except for *S*_20_, corresponding to the one described by Zhang et al. [29] and *S*_24_ reported by Wu et al. [30] and Gu et al. [31]. Another *S*-allele (present in ‘Cow-1’ and ‘Cow-2’) was found to have a previously non-described intron size pattern being named as *S*_22_, according to its homology with the NCBI GenBank accession HM053569, corresponding to the *S*_22_-*RNase* from the Chinese apricot cultivar ‘Tuxiangbai’. Lastly, two additional *S*-alleles, preliminary suggested as *S*_V_ and *S*_X_, were detected only once. The amplification of first *S*-*RNase* intron with the single primer pair SR1-F/SR1-R distinguished up to 13 *S*-alleles ranging from 260 (*S*_4_) to 427 bp (*S*_17_) (see Additional file 1: Table S1). Another primer combination (Pru-T2/SR1-R) allowed to amplify and distinguish two additional *S*-alleles *S*_6_ and *S*_24_. Exceptions were *S*_1_ and *S*_7_, and *S*_C_ and *S*_8_ pairs having exactly the same fragment sizes and *S*_19,_ *S*_20_ and *S*_X_ that could not be PCR-amplified with any of the four different primer combinations tested (data not shown). Sizes for the second *S*-*RNase* intron were approximately determined by agarose gel electrophoresis ranging from 300 (*S*_24_) to 2800 bp (*S*_C_) and allowed to define 15 *S*-alleles with a single primer pair (Pru-C2/Pru-C6R). Exceptions were *S*_5_ and *S*_6_, and *S*_8_ and *S*_C_ pairs as well as *S*_19_, *S*_20_ and *S*_V_ sharing similar fragment sizes, and *S*_3_ that could not be amplified with any of the two primer combinations used. Variability in size of the 5′-UTR *SFB* intron containing fragments ranged from 189 (*S*_17_) to 210 bp (*S*_1_) and facilitated the identification of 9 *S*-alleles. Exceptions were *S*_2_ and *S*_11_, *S*_18_ and *S*_22_, and *S*_C_ and *S*_8_ pairs sharing the same intron sizes and *S*_4_, *S*_5_, *S*_9_, *S*_19,_ *S*_20_, *S*_24,_ *S*_V_ and *S*_X_ which fragments could not be amplified. Altogether, combined data allowed us to distinguish unambiguously 18 out of the 20 *S*-alleles detected excluding *S*_V_ and *S*_X_ (see Additional file 1: Table S1). *S*_C_ and *S*_8_-alleles could only be distinguished by PCR-amplifying a fragment containing the 358 bp insertion present in *SFB*_C_ and absent in *SFB*_8_ [17, 18]. As a result, *S*_8_ was only detected in four Hungarian cultivars (‘Ceglédi óriás’, ‘Effect’, ‘Gönci Magyar’ and ‘Szegedi Mammut’) while *S*_C_ was found in 38 cultivars from many different origins (Table 3). Some already identified *S*-alleles (*S*_3_, *S*_20_ and *S*_24_) were not present in previously *S*-genotyped control cultivars (Table 3). For instance, putative *S*_3_ detected in ‘Harlayne’ and ‘Henderson’ has an estimated size for the first *S*-RNase intron that matches that of the ‘Sunglo’ *S*_3_-allele identified by Vilanova et al. [19] and nucleotide sequences from the three cultivars aligned by CLUSTALW showed 99% identity (data not shown). Moreover, according to the pedigree ‘Sunglo’ is the ‘Harlayne’ male parent [32]. *S*_20_ could not be amplified for the first *S*-RNase intron but the estimated size as well as the nucleotide sequence (99% identity) (see Additional file 2: Table S2) of the second one matches almost perfectly that of the *S*_20_-allele reported by Zhang et al. [29]. Interestingly, *S*_19_ could only be weakly amplified from ‘Mari de Cenad’ with PruC2/PruC6R primers and the fragment size observed in this work and that previously reported by Halázs et al. [20] is similar to *S*_20_, suggesting that both *S*-alleles might be the same. Unfortunately, this fragment could not be finally sequenced to confirm this point. *S*_24_ was only detected in ‘Ezzine’ but the estimated sizes for the first and second *S*-RNase introns, and the nucleotide sequence of the first one (248 bp), matches that *S*_24_-allele reported by Gu et al. [31] (see Additional file 2: Table S2).

### *M*-locus genotyping

**Fig. 1:**
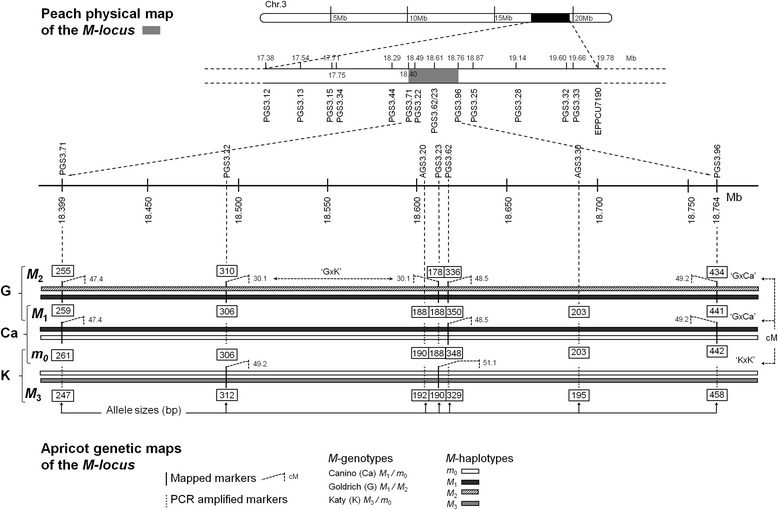
Apricot *M*-locus haplotypes structure. The *peach* syntenic region at the distal end of chr. 3 (*black box*) comprising the *M*-locus (*grey box*) is zoomed twice and shows SSRs (*dashed lines*) PCR-amplified in apricot cultivars ‘Goldrich’ (G), ‘Canino’ (Ca) and ‘Katy’ (K). SSR positions in *peach* genome (Kb) and allele sizes (bp) determined in apricot are indicated. *White, black, diagonal striped* and *grey thick line*s represent apricot *m*_0_, *M*_1_, *M*_2_ and *M*_3_ haplotypes, respectively. SSR anchoring positions are shown in centimorgans (cM) (*boxed numbers*) according to the available mapping populations (‘G × Ca’, ‘K × K’ and ‘G × K’)

The apricot *M*- and *M*’-loci were previously identified in cultivars ‘Canino’ and ‘Katy’ as carrying *S*-locus unlinked PPMs (*m* and *m*’, respectively) conferring SC [25, 26]. Up to 35 SSRs were found in the peach syntenic region of the *M*-locus (defined according to the ‘Canino’ genetic map) but just a five of them could be finally mapped due to several reasons (i.e. multiloci patterns, monomorphism, non- amplification, etc.). Two F_1_ populations segregating for the PPMs obtained by crossing the self-incompatible cultivar ‘Goldrich’ with ‘Canino’ (‘G × Ca’) and ‘Katy’ (‘G × K’) were used for mapping [25, 26]. Three SSRs were mapped using ‘G × Ca’ (PGS3.71, PGS3.62 and PGS3.96) and two with ‘G × K’ (PGS3.22 and PGS3.23). In this work, linkage phases were determined by using JoinMap 3.0 software [33] and SSR-alleles in coupling were subsequently used to define haplotypes for the *M*-locus in ‘Goldrich’, ‘Canino’ and ‘Katy’. These 5 SSR markers were also PCR-amplified from *M*- and *M*’-loci homozygote individuals derived from the self-pollination of ‘Canino’ [CC-77 (*MM*) and CC-67 (*mm*)] and ‘Katy’ [K06–17 (*m*’*m*’)] confirming estimated linkage phases and adding information to complete the haplotypes. Lastly, two additional SSRs identified from apricot genomic sequences located at the *M*-locus (AGS.20 and AGS.30) were also incorporated using the same procedure. SSR heterozygosity was estimated according to Nei [34] (Fig. 1; see Additional file 3: Table S3). As first finding, all SSR-alleles in coupling with the ‘Canino’ PPM *m* were found to be also in coupling with the ‘Katy’ PPM *m*’. In other words, both cultivars share the same pollen-part mutated haplotype designated hereinafter by *m*_0_. As a whole, these results lead to define initially four different *M*-haplotypes (*m*_0_, *M*_1_, *M*_2_ and *M*_3_) according to the genotypes established for ‘Canino’, ‘Katy’ and the reference cultivar ‘Goldrich’ (Fig. 1).

These seven SSR markers were subsequently PCR-genotyped in the remaining accessions. Structures of up to 34 additional *M*-haplotypes were then statistically inferred (see Methods for details) while *m*_0_, *M*_1_, *M*_2_ and *M*_3_ were fully confirmed (see Additional file 4: Table S4). For the sake of simplicity and to facilitate the graphical representation of their relative frequencies, *M*-haplotypes were grouped into ‘main classes’ when they differ in no more than three SSR alleles (resulting in a total of 20 *M*-haplotype ‘main classes’: *m*_0_ and *M*_1_ to *M*_19_). Thus, *M*-haplotypes were designed by two sub-indexes, the first one corresponds to the ‘main class’ and the second defines the subtype (see Additional file 4: Table S4). Structures of the *M*-haplotypes corresponding to the cultivars ‘Kech-pshar’ and ‘Fergani’ could not be inferred but according to the SSR allele sizes all four are distinct (data not shown). Phylogenetic reconstructions from Jaccard’s and Bruvo’s distances show that some ‘main classes’ clustered close together suggesting common ancestry such as, for instance, *M*_7_ and *M*_8_; *M*_2_, *M*_4_ and *M*_16_ or *m*_0_, *M*_1_ and *M*_13_ (Fig. 3). In this latter group, the nearest haplotypes to the majority haplotype *m*_0–0_ were *M*_1–2_, *M*_1–1_ and *M*_1–0_ showing Bruvo’s distance values of 0.18, 0.22 and 0.26, respectively.

### Mutations conferring self-compatibility in apricot

The *S*_C_-haplotype known to confer SC [17] was found in 38 accessions (being homozygous in 7) (Table 3). Thirty out of the 38 were self-compatible, 6 have undetermined phenotype and two were male-sterile. The *m*_0_-haplotype associated with SC by Zuriaga et al. [25, 26] was detected in a total of 19 accessions (being homozygous in 8): 8 were previously shown to be self-compatible, 5 were undetermined and one was classified as self-incompatible (‘Cow-2’) since it did not produce any fruit after self-pollination (Tables 2 and 3). As a whole, all self-compatible accessions analyzed have at least one of these two haplotypes already known to confer SC (*S*_C_ and/or *m*_0_) except for ‘Harlayne’, ‘Henderson’, ‘Shalah’ and ‘Mariem’. The number of self-compatible haplotypes carried by each accession varied from 0 to 4. A total of 21 accessions do not carry neither *S*_C_ nor *m*_0_, 26 carry only one self-compatible haplotype (corresponding to *S*- and *M*-genotypes *S*_C_/x or *m*_0_/x), 13 carry up to two (*S*_C_/*S*_C_, *m*_0_/*m*_0_ or *S*_C_/x-*m*_0_/x), 1 carry three (i.e. Gavatxet *S*_20_/*S*_C_-*m*_0_/*m*_0_) and 6 carry four (*S*_C_/*S*_C_-*m*_0_/*m*_0_) (Table 3).

Segregation of the *S*-genotypes in self-progenies obtained from two self-compatible cultivars carrying a single copy of the *m*_0_-haplotype (‘Portici’ and ‘Corbató’) supported a mutation outside the *S*-locus as the cause for the phenotype (Table 4). Progenies from self-compatible cultivars (‘Dulcinea’ and ‘Bebecou’) not carrying the *m*_0_-haplotype were used as controls to confirm this finding (Table 4). Similar results were observed in other accessions (with fewer offspring) carrying (‘Cow-1’ and ‘Cristalí’) and not carrying (‘Ezzine’ and ‘Ninfa’) the *m*_0_-haplotype (data not shown). Moreover, distortion ratios detected in the segregation of SSR markers tightly linked to the *M*-locus (AGS.20 and PGS3.23) point out that the mutation is located at the *M*-locus (Table 4).

**Table 4:** Segregation of *S*-*RNase* alleles in controlled self-pollinations

Cultivar *S*-genotype	Progeny *S*-genotypes			Total^a^	Ratio 1:1^b^	Ratio 1:2:1^b^	Ratio 1:1^c^
	A	H	B				
Bebecou (*S*_6_/*S*_C_)	0 (*S*_6_/*S*_6_)	33 (*S*_6_/*S*_C_)	45 (*S*_C_/*S*_C_)	96 (78)	1.85 (0.17)	53.8 (0)	n.d.^d^^.^
Dulcinea (*S*_C_/*S*_2_)	36 (*S*_C_/*S*_C_)	37 (*S*_C_/*S*_2_)	0 (*S*_2_/*S*_2_)	104 (73)	0.34 (0.56)	37.9 (1e-8)	n.d.
Corbató (*S*_5_/*S*_2_)	10 (*S*_2_/*S*_2_)	17 (*S*_5_/*S*_2_)	9 (*S*_5_/*S*_5_)	44 (36)	---	0.17 (0.92)	0.17 (0.68) [24]
Portici (*S*_2_/*S*_20_)	16 (*S*_2_/*S*_2_)	24 (*S*_2_/*S*_20_)	4 (*S*_20_/*S*_20_)	59 (44)	---	6.91 (0.03)	0.90 (0.35) [79]

^a^Number of individuals from the self-progeny and total analyzed (between parentheses) are indicated

^b^χ^2^ and (*P*) values for *S*-genotypes expected ratios considering a single mutation unlinked (1:2:1) or linked to the *S*-locus (1:1)

^c^χ^2^ and (*P*) values for PGS3.23 (in ‘Corbató’ self-progeny) and AGS.20 (in ‘Portici’ self-progeny) genotypes expected ratios considering a single mutation located at the *M*-locus (1:1). No homozygote for the SSR-allele in repulsion-phase linkage with the *m*_0_-haplotype was detected in either case. [*n*] indicates number of individuals tested

^d^n.d. Not determined

Interestingly, *S*-genotypes segregation found in the ‘Portici’ progeny might also indicate the presence of another mutation affecting the *S*_2_-haplotype, since the number of *S*_2_ *S*_2_ genotypes was unexpectedly high (Table 4). Analysis of genomic DNA fragments containing the complete sequence of *S*_2_-*RNase* and *SFB*_2_ alleles from ‘Portici’ revealed only one mismatch (A/G) within *SFB*_2_ located at position 1.296. This change leads to a non-synonymous substitution (lysine by arginine) in the hypervariable region HVb.

Lastly, *S*- and *M*-genotyping of ‘Canino’ selected clonal sibs (14–4, 14–6 and 9–7) [35] did not reveal differences with ‘Canino’ (Table 3), pointing out that *S*_C_ and *m* mutations are shared by all these four accessions.

### Geographical distribution of self-(in)compatibility

**Fig. 2:**
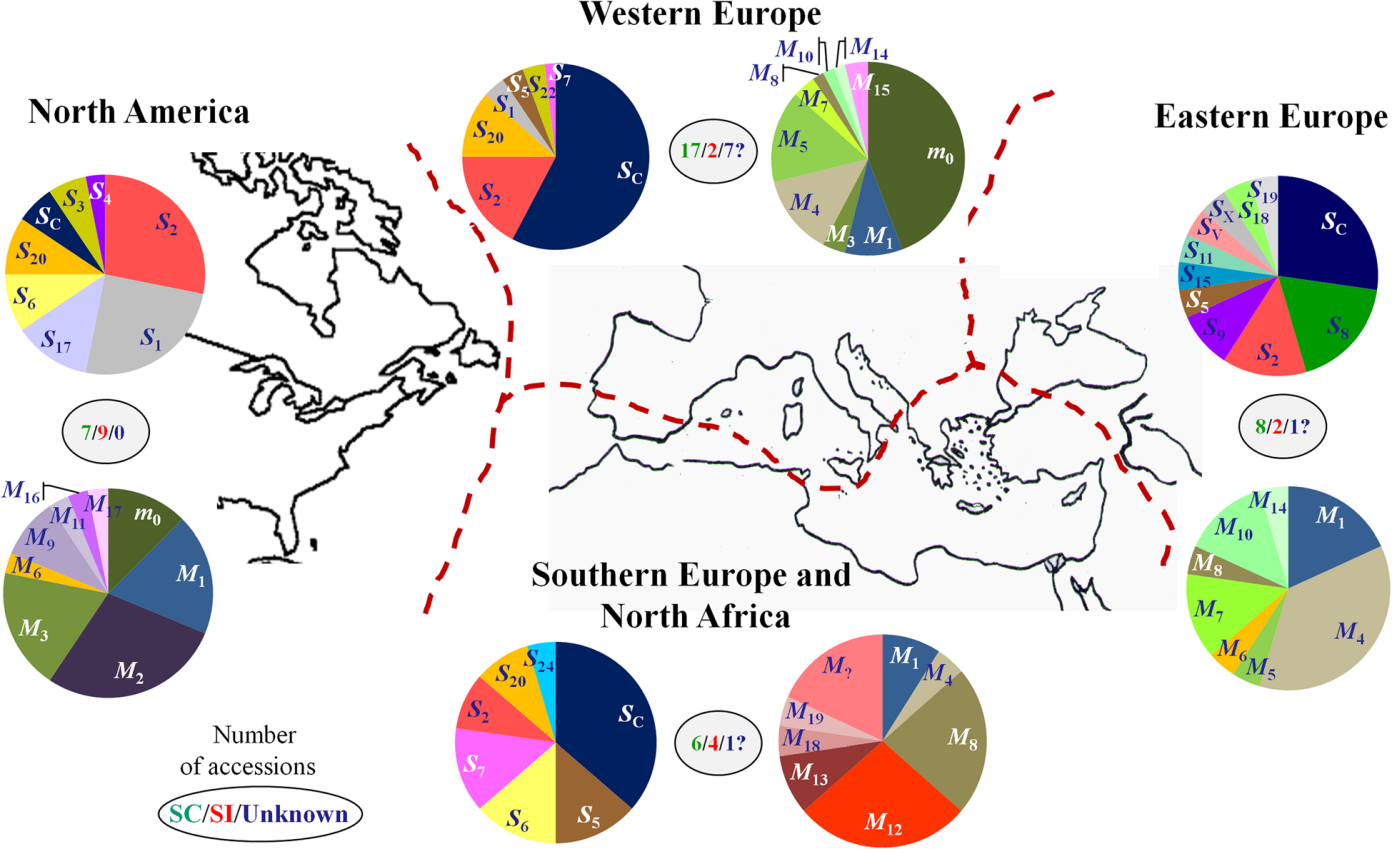
*S*- and *M*-locus haplotypes distribution according to geographic areas. Apricot accessions analyzed in this study were grouped in four arbitrarily defined geographic areas represented by the *bottom map*: Western Europe (WE), North America (NA), Southern Europe and North Africa (SE/NAf) and Eastern Europe (EE). Accordingly, relative frequencies for *S*- and *M*-haplotypes (pie charts) are shown for each area (clonal sibs from ‘Canino’ were excluded from estimations). For the sake of simplicity *M*-locus haplotypes are represented in their ‘main classes’. *Question marks* (?) designate not defined haplotypes. Number of accessions showing self-(in)compatible phenotype are encircled (*green* color means SC, *red* SI and *blue* undetermined phenotype)

For the sake of the analysis, cultivars and accessions studied in this work were grouped as belonging to four big geographic areas according to the country of origin, pedigree (Table 1) and data about dissemination [36–38]: North America (NA), Western Europe (WE), Eastern Europe (EE) and Southern Europe/North Africa (SE/NAf). Nonetheless, this classification is obviously arbitrary and exhibits some inconsistencies. This is particularly true for the WE and SE/NAf groups considered separately but clearly interrelated. Attending to geographic criteria, cultivars from Italy, France and Spain (Valencia region) were grouped into the first one while those from Tunisia, Greece and Spain (Murcia region) into the second. Spanish cultivars were divided into two subgroups that might reflect some differences according to [49]. Parentage relations among some cultivars from WE and SE/NAf groups (i.e. ‘Canino’ with ‘Ouardi’ and ‘Sayeb’) underscore the inaccuracy of this classification but, assuming these limitations, it produces more balanced groups without affecting data interpretation. Thus, SC phenotype is distributed across all four groups but frequencies varied from 2/19 (SI/SC) in WE to 9/16 in NA (Fig. 2). Similarly, blooming time types from early/mid-early to mid-late/late are present in all four groups, but frequency ranged from 9/24 in WE to 1/16 in NA (Table 1). In general it could be said that most early/mid-early classified accessions are self-compatible (13/15) but this phenotype is also generally found in many mid-late/late classified accessions (12/17). Therefore, even considering the reduced number of scored accessions and the limited phenotype data, it seems that there is no correlation between SC and early blooming in cultivated apricots.

Frequencies of the *S*-alleles also varied between the different regions analyzed. For instance, *S*_2_ and *S*_C_ are present in all four groups but, on the contrary, *S*_8_ and *S*_9_ are only present in EE, *S*_3_ in NA and *S*_7_ in SE/NAf (Fig. 2). Regarding *S*-alleles distribution it is also important to take into account synonymies and homonymies. In the first case, BLASTN has revealed that some of the *S*-alleles initially found and numbered from European accessions have also been detected in Chinese cultivars but named differently. For instance, a 659 bp fragment containing the second intron of the *S*_17_-*RNase* present in the North-American cultivar ‘Aurora’ (as well as in ‘SEO’, ‘Orange Red’ and ‘Henderson’) shows >99% identity with those from *S*_44_ and *S*_9_-*RNases* identified from the Chinese cultivars ‘Shailaiyulvke’ and ‘Xinshiji’, respectively, suggesting these three *S*-alleles to be the same. Similar examples are reported for *S*_1_, *S*_4_, *S*_9_, *S*_12_, *S*_16_ and *S*_20_-*RNases*. The other way around, BLASTN also points out homonymies. For example, *S*_9_ derived from the Hungarian cultivar ‘Ceglédi óriás’ is different to that *S*_9_ reported from the Chinese cultivar ‘Xinshiji’ (see Additional file 2: Table S2). BLASTN has also detected significant similarities between *S*_13_ reported in accessions from Armenia (including ‘Shalah’), Turkey, Tunisia and Morocco [20–22] and *S*_5_, previously found in Southern Spanish cultivars [19], and among *S*_12_, detected in Turkey and Tunisia [20, 22], *S*_24_ and *S*_4_ (see Additional file 2: Table S2).

**Fig. 3:**
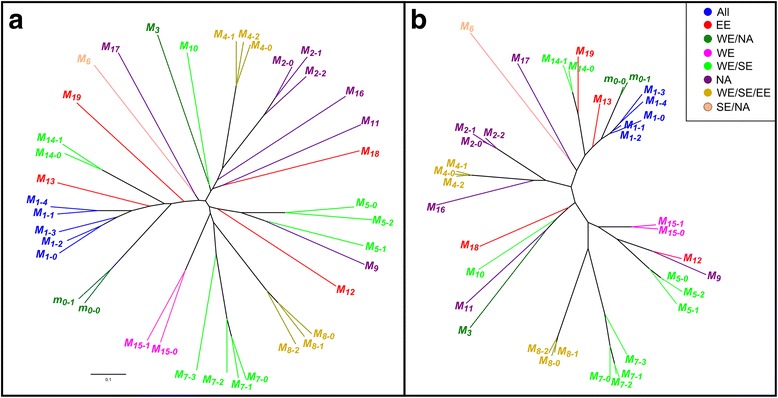
Clustering analysis of apricot *M*-locus haplotypes based on genetic distances**. a** Clustering obtained by Neighbor-Joining algorithm using Jaccard’s distance. **b** Clustering obtained by Neighbor-Joining algorithm using Bruvo’s distance. Colors represent geographic areas where the distinct *M*-locus haplotypes were detected (see legend)

Distribution of the 38 inferred *M*-haplotypes is not uniform either. The *M*_1_ ‘main class’ is present in all four groups, *m*_0_ is only found in WE and NA and others, such as *M*_12_ and *M*_2_, were exclusive for EE and NA groups, respectively (Figs. 2 and 3).

## Discussion

### *S*-alleles and the apricot diffusion from Armenian and Chinese centers to Europe

Most apricot *S*-alleles identified in this work had been previously reported except for *S*_22_, *S*_V_ and *S*_X_ [19, 27–30]. Geographical distribution of these *S*-alleles is heterogeneous including a few widely disseminated and others restricted to certain areas. These results are in agreement with previous works. For instance, *S*_2_ and *S*_C_ had already been found in accessions from all countries analyzed in this work but also in Turkey [20], Morocco [21], Afghanistan [18], Iran [40], etc. On the contrary, *S*_1_ is only present in NA and in a few accessions from WE. Meanwhile, *S*_10_ to *S*_14_-alleles, suggested to be from Armenian origin by Halázs et al. [20], have been detected in Turkey, Tunisia, Morocco and in a few materials from EE but they were thought to be absent in WE [20–22, 41]. However, *S*_5_ suggested to be the same that *S*_13_ connects (along with *S*_6_) Armenian, Eastern-Turkish and Moroccan accessions [20–22] with Southern-Spanish accessions [19, 42] supporting the apricot Southwest-Mediterranean diffusion route, from the Irano-Caucasian gene pool, proposed by Bourguiba et al. [38].

The heterogeneous distribution of the apricot *S*-alleles is a consequence of the crop domestication and diffusion and this process has also produced a genetic bottleneck detected in neutral SSR markers [38]. Though not quantified this loss of genetic diversity can also be grossly appreciated at the *S*-locus. For instance, China is not only considered a center of origin but also a probable domestication center for the species [38] and at least 20 different *S*-alleles have been found in just 30 Chinese cultivars [29, 30]. This variability is considerably much higher than that detected in European accessions and, interestingly, only 5 of these *S*-alleles (*S*_9_, *S*_14_, *S*_17_, *S*_20_ and *S*_24_) seem to be represented in apricot cultivars outside China. In addition, other *S*-alleles detected in far-east region cultivars such as *S*_15_/*S*_18_ in ‘Kech-pshar’ (Uzbekistan), *S*_10_ in ‘Harmat’ (Armenia) [27] and *S*_V_/*S*_X_ in ‘Fergani’ (former USSR) are not found in European accessions. *S*-alleles can therefore be a useful tool to study apricot dissemination but the establishment of a unified nomenclature would be advisable to favor this goal.

### Pollen-part mutated *m*-haplotype origin and dissemination

The two *S*-locus unlinked PPMs (*m* and *m*’) conferring SC in apricot cultivars ‘Canino’ and ‘Katy’ were found to be located in an overlapping region at the distal end of chr. 3 [26]. This finding suggested that both PPMs might be the same. However, in addition to their different geographic origins, ‘Canino’ and ‘Katy’ exhibited a high value for the Nei’s genetic distance (0.83) estimated on the basis of 85 SSR markers distributed across the whole genome [26]. Thus, initially it was hypothesized that these two PPMs originated independently but might affect the same gene. Nevertheless, a more detailed analysis of a set of SSR markers linked to the *M*-locus has allowed us to determine that haplotypes in coupling with the PPMs have the same structure in both cultivars. Thus, pollen-part mutated *m*- and *m*’-haplotypes previously associated with SC in ‘Canino’ and ‘Katy’ cultivars [25, 26] are now considered to be the same (*m*_0_). Moreover, in this work the *m*_0_-haplotype has been detected in 17 additional accessions (excluding ‘Canino’ clonal sibs) mainly Spanish (12 in total) but also from USA, Australia, France and Italy. Fifteen of them were confirmed as self-compatible (exceptions were ‘Cow-2′ described above as well as ‘Gandía’, ‘Gavatxet’, ‘Manrí’ and ‘Martinet’ with undetermined phenotype). The *m*_0_-haplotype was frequently accompanied by the *S*_C_-allele in confirmed self-compatible accessions (7 cases), which might suggest that mutations conferring SC tend to accumulate once the system is broken due to relaxed selective constraints [43]. Following this reasoning and considering the higher prevalence of the *S*_C_-allele in confirmed self-compatible accessions (74%) compared with the *m*_0_-haplotype (23%), it might also be speculated that PPM *m* arose in a self-compatible accession carrying *S*_C_. However, against this hypothesis is the fact that the *m*_0_-haplotype alone was also found in 6 confirmed self-compatible accessions and, therefore, further analyses are necessary to clarify this point. This latter group includes ‘Portici’ (*S*_2_/*S*_20_-*M*_1–4_/*m*_0–0_), previously shown to lack the *S*_C_-haplotype but which causative mutation was still unknown [44].

Beside the *m*_0_-haplotype, 37 additional *M*-haplotypes were identified by SSR analysis being grouped in 19 ‘main classes’. Microsatellite haplotype distances were analyzed by two alternative clustering methods to validate results. The first one relies on the proportion of shared alleles assuming independence and ignoring mutational processes which can bias distances, particularly when loci are highly polymorphic. This method was based on the similarity coefficient for binary data developed by Jaccard [45]. The second takes into account the stepwise mutation model considering higher likelihood for small than for large changes in microsatellite repeat number and it is based on the Bruvo’s distance [46]. Results obtained with both methods were equivalent but the second draws a more accurate phylogeny, where ‘main classes’ *m*_0_, *M*_1_ and *M*_13_ group close together suggesting a not too distant common ancestor. Remarkably, the closest haplotypes to *m*_0–0_ (*M*_1–0_, *M*_1–1_ and *M*_1–2_) are widely distributed geographically, while *m*_0–0_ seems to be restricted to NA and WE, and *M*_13_ to EE. The situation is different for the mutated *S*_C_-allele present in all geographic areas studied [18–21] whereas the ancestor *S*_8_-allele was only detected in Hungarian and Turkish cultivars [20]. Altogether, these results suggest that the *m*_0_-haplotype arose later in time than *S*_C_ since its distribution is much more limited. It can also be hypothesized that the last common ancestor for *m*_0_, *M*_1_ and *M*_13_ spread throughout different regions and mutated somewhere in WE. Nevertheless, in general, it has not been observed a significant correlation between geographic distribution and clustering of the *M*-haplotypes (see Fig. 3), and this may be due to the frequent crosses between genotypes from different groups [39].

### SC and genotyping: uncovering accessions carrying unanalyzed mutations

Part of the materials analyzed in this work could not be self-pollinated because adult trees were not available. However, though phenotype could not be directly assessed in these cases, according to the almost perfect association observed between SC and *S*_C_/*m*_0_-alleles it should be expected that most, if not all, accessions carrying whatever of these two alleles were self-compatible. However, some incongruities between phenotype and genotype need to be highlighted. For instance, in spite of having the *m*_0–0_ haplotype self-pollination of ‘Cow-2’ (*S*_20_/*S*_31_ *-M*_1–0_/*m*_0–0_) did not produce any fruit. This behavior could be due to fruit-set problems, as exemplified by the self-compatible cultivar ‘Búlida’ [47] which setting was also nearly null. Alternately, it may also be possible that *m*_0–0_ in ‘Cow-2’ does not carry the expected PPM but further analyses are needed to check this point. On the contrary, four cultivars classified as self-compatible do not carry neither the *S*_C_ nor the *m*_0–0_ haplotypes: ‘Mariem’ (*S*_7_/*S*_20_-*M*_1–0_/*M*_8–2_), ‘Shalah’ (*S*_5_/*S*_11_-*M*_8–1_/*M*_19_), ‘Harlayne’ (*S*_3_/*S*_20_-*M*_2–0_/*M*_9_) and ‘Henderson’ (*S*_3_/*S*_17_-*M*_2–0_/*M*_16_). Again, erroneous phenotyping can lead to discrepancies. In fact, ‘Mariem’ progeny could not be fully confirmed as derived from self-pollination (Table 2), ‘Shalah’ was reported as self-compatible by Burgos et al. [47] but scored as self-incompatible by Halázs et al. [20] and information describing ‘Henderson’ as self-compatible just comes from nursery catalogs. So, phenotype can be questioned in all these three cultivars. However, SC in ‘Harlayne’ is fully confirmed since it has been tested by several independent sources [47, 48]. Regarding genotypes, all the *S*-alleles carried by these cultivars can be frequently found in self-incompatible accessions and therefore expected to be ‘functional’. Something similar happens with *M*_1–0_ and *M*_2–0_ haplotypes but *M*_8–1_ and *M*_8–2_ belong to the *M*_8_-haplotype ‘main class’ always detected in self-compatible accessions (a total of 7 in this work and mostly East-European). However, no progenies are available and therefore it could not be tested whether *M*_8_ is mutated (non-functional) or not. As for *M*_16_ and *M*_19_, they were detected only once in the set of accessions analyzed. Overall, there are still no evidence supporting the nature of the mutation/s conferring SC in these cases and neither S/M-loci unlinked mutations nor other mutations affecting *S*- and *M*-loci can be discarded.

Lastly, genetic analysis suggests the presence of a SNP mutation within *SFB*_2_ HVb region in ‘Portici’ that could also be associated with SC. It could be speculated that a single non-synonymous change within a SFB hypervariable region might alter its specificity, since these domains (strongly hydrophobic and under positive selection) were already suggested to have a role in the specific recognition of S-RNases [49]. Interestingly, this change has also been found in ‘Katy’ *SFB*_2_ where a similar distortion was detected in the self-progeny [26]. However, further research is needed to validate this observation and other possibilities cannot be discarded (i.e. mutations affecting pollen viability).

### Forces selecting for self-compatibility in apricot

SC is therefore quite common in apricot but distribution is not uniform across geographic areas. SI is the prevalent phenotype in three out of the four major eco-geographical groups for apricot (centers of origin): Central Asian, Irano-Caucasian and Dzhungar-Zailij and also in the later proposed Chinese group [36]. In fact, studies about Chinese apricots do not report self-compatible cultivars [29, 30, 50]. On the contrary, SC predominates in the European group but some disequilibrium can also be observed. Among the materials analyzed in this work, SI is frequent in commercial North-American cultivars (two thirds) but unusual in West and East-European countries (one fifth). This might point out a non-European SI ancestral donor for North-American cultivars apricots as previously suggested for PPV resistance trait [51].

It is generally accepted that apricot genetic diversity decreases from east to south-west [36] and, in this context, it is questionable whether SC might be one of the causes. Recent works suggest that a substantial part of this loss is independent of the selection impact due to the domestication bottleneck, being more related to apricot diffusion routes [38]. In general, self-fertility would be advantageous under unfavorable pollination conditions including early blooming [52] but this correlation has not been observed in this study. However, we cannot discard that SC was originally selected from early-flowering apricot types. In fact, in cherry (*Prunus avium*) only a very few cultivars are reported to be naturally self-compatible and these include two exceptionally early-flowering cherries, ‘Cristobalina’ and ‘Kronio’ [53, 54]. Moreover, they were selected independently since each one carry a different mutation conferring SC, ‘Cristobalina’ in a non-S-locus pollen modifier [24] and ‘Kronio’ in the *SFB*_5_ allele [54]. On the other hand, in spite of the benefits linked to SC for growing stone fruits (increased yield, removing of interspersed pollinators, etc.) it was not usually considered as a target trait. Some morphological and agronomic traits are putatively linked to the *S*- (fruit shape) and *M*-loci (polycarpel and flower color) in *Prunus* [55] but it seems unlikely that SC was brought in by linkage drag with these particular traits. Thus, SC in apricot is more likely the indirect result of selective breeding on major traits, such as ecological adaptation [36], and only recently it has become a breeding objective by itself. In any case, SC must have had an effect on reducing diversity since *S*_C_ *S*_C_ homozygote cultivars (potentially resulting from self-pollination events) are frequently found in most European countries.

In this work we have confirmed two independent PPMs (*S*_C_ and *m*) as the main causes for SC in apricot. According to [16] a total of 29 mutations conferring SC have been identified in *Prunus*. Most of them affect *S*-locus (27) and only two correspond to mutated modifiers, the apricot mutated *m*_0_-haplotype and a non *S*-locus PPM in sweet cherry [17, 24]. Indeed, these two are located at the distal end of chr.3 but it is still unknown whether they affect different genes [26]. Within the supposedly complex GSI mechanism it is noteworthy this low number of mutations affecting modifiers. One possible explanation is that other modifiers participating in the control of the GSI system may be redundant and when mutated do not confer SC. Furthermore, loss of function of some factors might lead to SI as may be predicted for SLFL2 [14]. Lastly, in spite of the high number of accessions already evaluated in all *Prunus* species, it can also be argued that a more exhaustive screening might reveal novel mutations. Indeed, in the light of the results of this work, it is conceivable that additional mutations conferring SC may remain ‘hidden’ in the large amount of unanalyzed plant material.

## Conclusions

It has been shown that apricot *S*-allele geographical distribution is heterogeneous. In addition, it seems that the loss of genetic diversity, hypothesized for this crop during the dissemination process, affected not only neutral markers but also the *S*-locus. Indeed, only a few *S*-alleles present in self-incompatible cultivars from China (the domestication center of the species) can be found in European cultivars. However, *S*_C_, one of the most diffused *S*-alleles and the main cause of SC in apricot has not been detected in China. Apart from the PPM associated to *S*_C_, other *S*-locus unlinked PPMs conferring SC have been found in apricot cultivars ‘Canino’ and ‘Katy’. At odds with the initial hypothesis, we have shown that both are associated with the same *m*_0_-haplotype, in spite of the genetic distance between the two cultivars. Moreover, this haplotype has been surprisingly detected in other Spanish, French, Italian and North-American accessions. Its origin is still a matter of speculation but the phylogenetic analysis supports that *m*_0_ arose later in time than *S*_C_ from a widely distributed *M*-haplotype_._ As a whole, self-(in)compatibility in apricot seems to rely mainly on the *S* and *M* loci. However, other self-compatible mutants putatively carrying different mutations have been identified in this work. These results deepen our knowledge on the self-(in)compatibility trait in stone fruits and open new questions that deserve future research.

## Methods

### Plant Material and self-pollination test

Sixty seven apricot cultivars and accessions from diverse geographic origins were used in this study (Table 1). Most are currently kept at the collection of the *Instituto Valenciano de Investigaciones Agrarias* (IVIA) in Valencia (Spain). Part of this collection was kindly provided by *Frutales Mediterráneo S.A.* (FM) company, by the *Consellería de Agricultura, Pesca y Alimentación* (CAPA) and by the *Ministerio de Agricultura, Alimentación y Medio Ambiente* of Spain (MAGRAMA). Other samples, already used in previous works [56], were provided by the *Departamento de Mejora y Patología Vegetal del* CEBAS-CSIC in Murcia (Spain) and by the University of St. Istvan (Budapest, Hungary).

Trees from different cultivars and accessions (Table 2) were tested for SC by self-pollination in the field. Before anthesis, insect-proof bags were put over several branches, containing approximately 200–250 flower buds in total per cultivar *ad minimum*, to prevent cross pollination. Subsequent fruit set was recorded and fruits collected about 3 months later. Seed-derived embryos were dissected from the rest of the seed tissue and stored at −20 °C.

### DNA extraction

Two leaf discs were collected from each accession, frozen in liquid N_2_ and stored at −80 °C before DNA isolation. Genomic DNA was extracted following the method of [57] with slight modifications: the ratio of fresh leaf tissue/CTAB buffer was reduced to 10 mg/250 μl and nucleic acids were recovered by precipitation with 1× volume of isopropanol. DNA quantification was performed by NanoDrop ND-1000 spectrophotometer (Thermo Fisher Scientific, Wilmington, DE) and integrity was checked on 1% agarose gel by comparison with lambda DNA (Promega, Madison, WI, USA). Embryo DNA was extracted by incubating for 10 min at 95 °C with 20 μL of TPS (100 mM Tris-HCl, pH 9.5; 1 M KCl; 10 mM EDTA) isolation buffer [58].

### *S*-locus genotyping

Apricot *S*-alleles were identified by PCR-amplifying fragments comprising first and second introns of the *S*-*RNase* as well as the 5′-UTR *SFB* intron, respectively. Genomic DNA isolated from cultivars and accessions listed in Table 1 was used as PCR template. PCRs were performed in a final volume of 20 μL containing 1 × DreamTaq buffer, 0.2 mM of each dNTP, 20 ng of genomic DNA and 1 U of DreamTaq DNA polymerase (Thermo Fisher Scientific, Waltham, MA, USA). Previously developed primers designed from conserved regions of *Prunus armeniaca S*-*RNase* genomic sequences, SRc-F and SRc-R [19, 58] and from *Prunus avium S*-*RNase*-cDNA sequences, Pru-T2 and Pru-C2R [59], were used to amplify the first intron (see Additional file 5: Table S5) in all four possible combinations. The amplification was carried out using a temperature profile with an initial denaturing of 95 °C for 2 min; 30 cycles of 95 °C for 45 s, 52 °C for 1 min and 72 °C for 1 min 30 s; and a final extension of 60 °C for 30 min (UNO96, VWR, Radnor, PA, USA). Each PCR was performed by the procedure of [60] using three primers: the specific forward primer with M13(−21) tail at its 5′ end at 0.4 mM, the reverse primer at 0.8 mM, and the universal fluorescent-labeled M13(−21) primer at 0.4 mM. Allele lengths were determined using an ABI Prism 3130 Genetic Analyzer with the aid of GeneMapper software, version 4.0 (Applied Biosystems, Foster City, CA, USA).

The second intron was amplified using two sets of primers designed from *Prunus avium S*-*RNase*-cDNA sequences [61]: Pru-C2/Pru-C4R and Pru-C2/Pru-C6R (see Additional file 5: Table S5). PCRs were performed using 0.25 μM of each primer and the program previously described by [62] to amplify long PCR products. PCR products were electrophoresed in 0.8% (*w*/*v*) agarose gels using 1 x TBE (89 mM Tris, 89 mM boric acid, and 2 mM EDTA (pH 8.0)) buffer, stained with RedSafe Nucleic acid Staining Solution (iNtRON Biotechnology, Korea) and visualized under UV light. Molecular sizes of amplified fragments were estimated using GeneRuler 100 bp Plus DNA ladder (Thermo Fisher Scientific).

The 5′-UTR *SFB* intron was also amplified using the degenerate primer pair (F-BOX5′A/F-BOXintronR) developed by Vaughan et al. [63] from sweet cherry sequences (see Additional file 5: Table S5). PCR components, thermo-cycler conditions and detection procedure were identical to that described above for the first *S*-*RNase* intron. Two additional primers (RFBc-F/SFBins-R), designed from the consensus sequence of the *Prunus SFB* alleles [17], were used to amplify the *SFB*_C_ insertion from genomic DNA of several apricot cultivars in order to distinguish *S*_C_ and *S*_8_-alleles (see Additional file 5: Table S5).

### *M*-locus genotyping

Seven SSR markers comprised within (or flanking) the *M*-locus were genotyped: PGS3.71, PGS3.22, PGS3.62, PGS3.23 and PGS3.96 [25, 26] and AGS-20 and AGS-30 (this work) (see Additional file 3: Table S3). SSR amplifications were performed in a GeneAmp® PCR System 9700 thermal cycler (Perkin–Elmer, Freemont, CA, USA) in a final volume of 20 μL, containing 1 × DreamTaq buffer, 0.2 mM of each dNTP; 20 ng of genomic DNA and 1 U of DreamTaq DNA polymerase (Thermo Fisher Scientific). Regarding primers, PCRs were performed as described above by the procedure of [60]. The following temperature profile was used: 94 °C for 2 min, then 35 cycles of 94 °C for 45 s, 50–60 °C for 1 min, and 72 °C for 1 min and 15 s, finishing with 72 °C for 5 min. Allele lengths were determined using an ABI Prism 3130 Genetic Analyzer with the aid of GeneMapper software, version 4.0 (Applied Biosystems).

### Sequence analysis of PCR products containing *S*-*RNase* introns

PCR products containing the first and second introns of *S*_20_ and *S*_24_ *-RNase* alleles previously obtained from genomic DNA of ‘Ezzine’ (*S*_C_ *S*_24_), on one side, and from cultivars ‘Cow-2’ (*S*_20_ *S*_22_), ‘Harlayne’ (*S*_3_ *S*_20_), ‘Cristalí’ (*S*_20_ *S*_C_), ‘Gavatxet’ (*S*_20_ *S*_C_), ‘Mariem’ (*S*_20_ *S*_7_), ‘Perla’ (*S*_20_ *S*_2_), ‘Portici’ (*S*_20_ *S*_2_), ‘Stella’ (*S*_20_ *S*_6_), ‘Tadeo’ (*S*_20_ *S*_C_), ‘Veecot’ (*S*_20_ *S*_2_) and ‘Velázquez’ (*S*_20_ *S*_5_), on the other, were sequenced to check their identities. Similarly, PCR products associated with *S*_3_, *S*_17_ and *S*_22_ *-RNase* alleles obtained from cultivars ‘Harlayne’ (*S*_3_ *S*_20_), ‘Henderson’ (*S*_3_ *S*_17_), ‘Orange Red’ (*S*_6_ *S*_17_), ‘SEO’ (*S*_6_ *S*_17_), ‘Cow-1’ (*S*_1_ *S*_22_) and ‘Cow-2’ (*S*_20_ *S*_22_) were also sequenced. Primer combinations SRc-F/SRc-R and Pru-C2/Pru-C4R were used in all cases for the first and second introns respectively except for *S*_24_ (Pru-T2/SRc-R) and *S*_22_ (Pru-C2/Pru-C6R). PCR products were electrophoresed in 0.8% or 2% (*w*/*v*) agarose gels (second or first intron, respectively) stained with RedSafe Nucleic acid Staining Solution (iNtRON Biotechnology). Molecular sizes of the amplified fragments were estimated using GeneRuler 100 bp Plus DNA ladder (Thermo Fisher Scientific). Fragments were extracted and purified from the agarose gels using the Zymoclean Gel DNA Recovery kit (Zymo Research, Irvine, CA, USA). Sequences were determined automatically using an ABI PRISM 3100 Genetic Analyzer (Applied Biosystems) and the Big Dye Terminator Cycle Sequencing Kit v3.1 (Applied Biosystems) following the manufacturer’s instructions. Sequences were assembled and edited with the Staden package v.2.0 [64]. Multiple alignments were made with CLUSTALW v.1.82 [65] and edited in BioEdit v.7.0.9 [66]. Homology searches were performed against the NCBI Genbank database using the BLASTN program [67].

### *S*_2_-locus sequence analysis

Specific primers designed from the apricot *S*_2_-haplotype sequence [17] were used to amplify genomic fragments containing the complete *S*_2_-*RNase* (Sf-Hap2/Sr-Hap2) and *SFB*_2_ coding sequences (FBf-Hap2/FBr-Hap2) (see Additional file 5: Table S5) using ‘Portici’ (*S*_2_ *S*_20_) genomic DNA as template. PCR conditions and methods for isolating and sequencing (using the three additional primers SFBc-F, FBF5 and FBF6) these bands (see Additional file 5: Table S5) were the same reported above for fragments containing the *S*-*RNase* second intron.

### Clustering analysis

Reference *m*_0–0_ *, M*_1–0_ *, M*_2–0_ and *M*_3_-haplotypes were established using genetic maps from ‘G × Ca’, ‘K × K’ and ‘G × K’ populations through the automatic determination of linkage phases by JoinMap 3.0 [33]. Remaining *M*-haplotypes were inferred from SSR genotypes by comparing with the references and confirmed by using the EM algorithm [68] implemented in PowerMarker V3.25 software [69]. When available pedigree was used to confirm assignments. Similarities between *M*-haplotypes were estimated by using Jaccard’s similarity coefficient [45] through the Phylip 3.62 package [70] and Bruvo’s genetic distance [46] through a hand-made phyton script. Bootstrapped data matrices were obtained using the Phyltools 1.32 software [71] and used to test the stability of the neighbor joining trees constructed with the Phylip 3.62 package [70]. HyperTree software [72] was used to visualize the obtained trees.

## Additional files

Additional file 1: Table S1. Fragment sizes (bp) determined for *S-RNase* and *SFB* allele introns. Fragment sizes for the first *S-RNase* and the 5′-UTR *SFB* introns were exactly determined by an ABI PRISM 3100 Genetic Analyzer and those for the second *S*-*RNase* intron were approximately estimated from 0.8% agarose gels. References reporting *S*-allele molecular sizes for the first time are provided. (DOCX 15 kb)

Additional file 2: Table S2. Main results of homology searches performed by BLASTN against the NCBI Genbank database using *Prunus armeniaca S*-RNase sequences. Query sizes, Query cover %, Total score, E-value and Identity % are indicated. Putative names were assigned to the *S*-alleles according to the fragment sizes and sequence analyses. (DOCX 18 kb)

Additional file 3: Table S3. Characteristics of SSR primers developed from peach (PGS) and apricot genome sequences (AGS) located at the *M*-locus. Repeat motifs, Primer sequences, Scaffold positions, ORF (Prupe), Size range, N° of alleles and Heterozygosity (in the set of accessions analyzed) are indicated. (DOCX 14 kb)

Additional file 4: Table S4. Allele sizes (bp) for the SSR markers comprised into the *M*-haplotypes. ‘Main class’ as well as the subtype are indicated for every *M*-haplotype. Absolute frequency in the set of accessions analyzed is also provided. (DOCX 18 kb)

Additional file 5: Table S5. Primers used in this study. Sequences and references are indicated. (DOCX 14 kb)

## Abbreviations

EE: Eastern Europe
GSI: Gametophytic Self-Incompatibility
NA: North America
PPM: Pollen-Part Mutation
SC: Self-Compatibility
SE/NAf: Southern Europe/North Africa
SFB: *S*-locus F-box
SI: Self-Incompatibility
WE: Western Europe

## References

[1] Igic B, Kohn JR (2001). Evolutionary relationships among self-incompatibility RNases. Proc Natl Acad Sci U S A.

[2] De Nettancourt D (2001). Incompatibility and incongruity in wild and cultivated plants.

[3] McClure BA, Haring V, Ebert PR, Anderson MA, Simpson RJ, Sakiyama F, Clarke AE (1989). Style self-incompatibility gene products of *Nicotiana alata* are ribonucleases. Nature.

[4] Lai Z, Ma W, Han B, Liang L, Zhang Y, Hong G, Xue Y (2002). An F-box gene linked to the self-incompatibility (*S*) locus of *Antirrhinum* is expressed specifically in pollen and tapetum. Plant Mol Biol.

[5] Sijacic P, Wang X, Skirpan AL, Wang Y, Dowd PE, McCubbin AG, Huang S, Kao T-h (2004). Identification of the pollen determinant of S-RNase-mediated self-incompatibility. Nature.

[6] Ushijima K, Sassa H, Dandekar AM, Gradziel TM, Tao R, Hirano H (2003). Structural and transcriptional analysis of the self-incompatibility locus of almond: Identification of a pollen-expressed F-box gene with haplotype-specific polymorphism. Plant Cell.

[7] Hua Z, Kao T-h (2006). Identification and characterization of components of a putative *Petunia S*-locus F-box-containing E3 ligase complex involved in S-RNase based self-incompatibility. Plant Cell.

[8] Huang J, Zhao L, Yang Q, Xue Y (2006). AhSSK1, a novel SKP1-like protein that interacts with the S-locus F-box protein SLF. Plant J.

[9] Kubo K, Entani T, Tanaka A, Wang N, Fields AM, Hua Z, Toyoda M, Kawashima S, Ando T, Isogai A, Kao T, Takayama S (2010). Collaborative non-self recognition system in S-RNase-based self-incompatibility. Science.

[10] Fujii S, Kubo K, Takayama S (2016). Non-self- and self-recognition models in plant self-incompatibility. Nat Plants.

[11] Kakui H, Kato M, Ushijima K, Kitaguchi M, Kato S, Sassa H (2011). Sequence divergence and loss-of-function phenotypes of S locus F-box brothers (SFBB) genes are consistent with non-self recognition by multiple pollen determinants in self-incompatibility of Japanese pear (*Pyrus pyrifolia*). Plant J.

[12] Matsumoto D, Tao R (2016). Distinct self-recognition in the *Prunus* S-RNase-based gametophytic self-incompatibility system. Hort J.

[13] Tao R, Iezzoni AF (2010). The S-RNase-based gametophytic self-incompatibility system in *Prunus* exhibits distinct genetic molecular features. Sci Hort.

[14] Matsumoto D, Tao R (2016). Recognition of a wide-range of S-RNases by S locus F-box like 2, a general-inhibitor candidate in the Prunus-specific S-RNase-based self-incompatibility system. Plant Mol Biol.

[15] Yamane H, Tao R (2009). Molecular basis of self-(in)compatibility and current status of S-genotyping in Rosaceous fruit trees. J Jpn Soc Hort Sci.

[16] Hegedűs A, Lénárt J, Halász J (2012). Sexual incompatibility in Rosaceae fruit tree species: molecular interactions and evolutionary dynamics. Biol Plant.

[17] Vilanova S, Badenes ML, Burgos L, Martínez-Calvo J, Llácer G, Romero C (2006). Self-compatibility of two apricot selections is associated with two pollen-part mutations of different nature. Plant Physiol.

[18] Halász J, Pedryc A, Hegedűs A (2007). Origin and dissemination of the pollen-part mutated S_C_ haplotype which confers self-compatibility in apricot (*Prunus armeniaca*). New Phytol.

[19] Vilanova S, Romero C, Llácer G, Badenes ML (2005). Identification of self-(in)compatibility alleles in apricot by PCR and sequence analysis. J Am Soc Hortic Sci.

[20] Halász J, Pedryc A, Ercisli S, Yilmaz KU, Hegedűs A (2010). *S*-genotyping supports the genetic relationships between Turkish and Hungarian apricot germplasm. J Amer Soc Hort Sci.

[21] Kodad O, Hegedűs A, Socias i Company R, Halász J (2013). Self-(in)compatibility genotypes of Moroccan apricots indicate differences and similarities in the crop history of European and North African apricot germplasm. BMC Plant Biol.

[22] Lachkar A, Fattouch S, Ghazouani T, Halasz J, Pedryc A, Hegedűs A, Mars M (2013). Identification of self-(in)compatibility *S*-alleles and new cross-incompatibility groups in Tunisian apricot (*Prunus armeniaca* L.) cultivars. J Hortic Sci Biotechnol.

[23] McClure BA, Cruz-García F, Beecher B, Sulaman W (2000). Factors affecting inter- and intra-specific pollen rejection in *Nicotiana*. Ann Bot.

[24] Wünsch A, Hormaza JI (2004). Genetic and molecular analysis in Cristobalina sweet cherry, a spontaneous self-compatible mutant. Sex Plant Reprod.

[25] Zuriaga E, Molina L, Badenes ML, Romero C (2012). Physical mapping of a pollen modifier locus controlling self-incompatibility in apricot and synteny analysis within the Rosaceae. Plant Mol Biol.

[26] Zuriaga E, Muñoz-Sanz JV, Molina L, Gisbert AD, Badenes ML, Romero C (2013). An S-locus independent pollen factor confers self-compatibility in “Katy” apricot. PLoS One.

[27] Halász J, Hegedüs A, Hermán R, Stefanovits-Bányai E, Pedryc A (2005). New self-incompatibility alleles in apricot (*Prunus armeniaca* L.) revealed by stylar ribonuclease assay and S-PCR analysis. Euphytica.

[28] Halász J (2007). Molecular background of the S-locus controlled self-incompatibility in apricot.

[29] Zhang L, Chen X, Chen X, Zhang C, Liu X, Ci Z, Zhang H, Wu C, Liu C (2008). Identification of self-incompatibility (*S-*) genotypes of Chinese apricot cultivars. Euphytica.

[30] Wu J, Gu C, Zhang SL, Zhang SJ, Wu HQ, Heng W (2009). Identification of *S*-haplotype specific *S-RNase* and *SFB* alleles in native Chinese apricot (*Prunus armeniaca* L.). J Hortic Sci Biotechnol.

[31] Gu C, Wu J, Du YH, Yang YN, Zhang SL (2013). Two different *Prunus SFB* alleles have the same function in the self-incompatibility reaction. Plant Mol Biol Rep.

[32] Brooks RM, Olmo HP (1997). The Brooks and Olmo Register of Fruit and Nut Varieties.

[33] Van Ooijen JW, Voorrips RE (2001). JoinMap®3.0, Software for the calculation of genetic linkage maps.

[34] Nei M (1973). Analysis of gene diversity in subdivided populations. Proc Natl Acad Sci U S A.

[35] Orero G, Cuenca J, Romero C, Martínez-Calvo J, Badenes ML, Llácer G (2004). Selection of seedling rootstocks for apricot and almond. Acta Hort (ISHS).

[36] Mehlenbacher SA, Cociu V, Hough LF, Moore JN, Ballington JR (1990). Apricots. Genetic Resources of Temperate Fruit and Nut Crops.

[37] Faust M, Surányi D, Nyujtó F (1998). Origin and dissemination of apricot. Hort Rev.

[38] Bourguiba H, Audergon JM, Krichen L, Trifi-Farah N, Mamouni A, Trabelsi S, D’Onofrio C, Asma BM, Santoni S, Khadari B (2012). Loss of genetic diversity as a signature of apricot domestication and diffusion into de Mediterranean Basin. BMC Plant Biol.

[39] Martin C, Herrero M, Hormaza JI (2011). Molecular characterization of apricot germplasm from an old stone collection. PLoS One.

[40] Hajilou J, Grigorian V, Mohammadi SA, Nazemmieh A, Romero C, Vilanova S, Burgos L (2006). Self- and cross-(in)compatibility between important apricot cultivars in northwest Iran. J Hort Sci Biotech.

[41] García S, Sánchez-Capuchino JA, Dalmau F, Casanova R, Salazar D (1985). Taxonomía pomológica de los albaricoqueros valencianos (I). Agrícola Vergel.

[42] Burgos L, Pérez-Tornero O, Ballester J, Olmos E (1998). Detection and inheritance of stylar ribonucleases associated with incompatibility alleles in apricot. Sex Plant Reprod.

[43] Kimura M (1980). A simple method for estimating evolutionary rates of base substitutions through comparative studies of nucleotide sequences. J Mol Evol.

[44] Dondini L, De Franceschi P, Sansavini S (2012). Gametophytic incompatibility in pome and stone fruits: genes controlling *S*-locus. Acta Hort (ISHS).

[45] Jaccard P (1901). Étude comparative de la distribution florale dans une portion des Alpes et des Jura. Bull Soc Vaudoise Sci Nat.

[46] Bruvo R, Michiels NK, D’Souza TG, Schulenburg H (2004). A simple method for the calculation of microsatellite genotype distances irrespective of ploidy level. Mol Ecol.

[47] Burgos L, Alburquerque N, Egea J (2004). Review. Flower biology in apricot and its implications for breeding. Span J Agric Res.

[48] Milatović D, Nikolić D, Fotirić-Akšić M, Radović A (2013). Testing of self-(in)compatibility in apricot cultivars using fluorescence microscopy. Acta Sci. Pol. Hortorum Cultus.

[49] Ikeda K, Igic B, Ushijima K, Yamane H, Hauck NR, Nakano R, Sassa H, Iezzoni AF, Kohn JR, Tao R (2004). Primary structural features of the *S* haplotype specific F-box protein, SFB, in *Prunus*. Sex Plant Reprod.

[50] Jie Q, Shupeng G, Jixiang Z, Manru G, Huairui S (2005). Identification of self-incompatibility genotypes of apricot (*Prunus armeniaca* L.) by S-allele-specific PCR analysis. Biotechnol Lett.

[51] Zhebentyayeva TN, Reighard GL, Lalli D, Gorina VM, Krška B, Abbott AG (2008). Origin of resistance to plum pox virus in Apricot: what new AFLP and targeted SSR data analyses tell. Tree Genet Genomes.

[52] Lloyd DG (1992). Self- and cross-fertilization in plants. II. The selection of self-fertilization. Int J Plant Sci.

[53] Cachi AM, Hedhly A, Hormaza JI, Wünsch A (2014). Pollen tube growth in the self-compatible sweet cherry genotype, ‘Cristobalina’, is slowed down after self-pollination. Ann Appl Biol.

[54] Marchese A, Boskovic RI, Caruso T, Raimondo A, Cutuli M, Tobutt KR (2007). A new self-compatibility haplotype in the sweet cherry ‘Kronio’ *S*_5_′ attributable to a pollen-part mutation in the SFB gene. J Exp Bot.

[55] Dirlewanger E, Graziano E, Joobeur T, Garriga-Calderé F, Cosson P, Howad W, Arús P (2004). Comparative mapping and marker-assisted selection in Rosaceae fruit crops. Proc Natl Acad Sci.

[56] Romero C, Pedryc A, Munoz V, Llacer G, Badenes ML (2003). Genetic diversity of different apricot geographical groups determined by SSR markers. Genome.

[57] Doyle JJ, Doyle JL (1987). A rapid isolation procedure for small quantities of fresh leaf tissue. Phytochem Bull.

[58] Thomson D, Henry R (1995). Single-step protocol for preparation of plant tissue for analysis by PCR. BioTechniques.

[59] Romero C, Vilanova S, Burgos L, Martínez-Calvo J, Vicente M, Llácer G, Badenes ML (2004). Analysis of the *S*-locus structure in *Prunus armeniaca* L. Identification of *S*-haplotype specific *S-RNase* and *F-box* genes. Plant Mol Biol.

[60] Schuelke M (2000). An economic method for the fluorescent labelling of PCR fragments. Nat Biotechnol.

[61] Tao R, Yamane H, Sugiura A (1999). Molecular typing of *S*-alleles through identification, characterization and cDNA cloning for S-RNases in sweet cherry. J Am Soc Hortic Sci.

[62] Sonneveld T, Tobutt KR, Robbins TP (2003). Allele-specific PCR detection of sweet cherry self-incompatibility (S) alleles S1 to S16 using consensus and allele-specific primers. Theor Appl Genet.

[63] Vaughan SP, Russell K, Sargent DJ, Tobutt KR (2006). Isolation of S-locus F-box alleles in *Prunus avium* and their application in a novel method to determine self-incompatibility genotype. Theor Appl Genet.

[64] Bonfield J: Staden package, version 2.0. http://staden.sourceforge.net (2004). Accessed 16 September 2016.

[65] Thompson JD, Higgins DG, Gibson TJ (1994). CLUSTAL W: improving the sensitivity of progressive multiple sequence alignment through sequence weighting, positions-specific gap penalties and weight matrix choice. Nucleic Acids Res.

[66] Hall TA (1999). BioEdit: a user-friendly biological sequence alignment editor and analysis program for Windows 95/98/NT. Nucleic Acids Symp Ser.

[67] Altschul SF, Gish W, Miller W, Myers EW, Lipman DJ (1990). Basic local alignment search tool. J Mol Biol.

[68] Excoffier L, Slatkin M (1995). Maximum-Likelihood estimation of molecular haplotype frequencies in a diploid population. Mol Biol Evol.

[69] Liu K, Muse SV (2005). PowerMarker: an integrated analysis environment for genetic marker analysis. Bioinformatics.

[70] Felsenstein J (2005). PHYLIP (Phylogeny Inference Package) version 3.6.

[71] Buntjer JB (1997). Phylogenetic computer tools (PhylTools). Version 1.32 for Windows.

[72] Bingham J, Sudarsanam S (2000). Visualizing large hierarchical clusters in hyperbolic space. Bioinformatics.

[73] Russell D (1998). The stonefruit cultivar system (A database of worldwide stonefruit cultivars and rootstocks).

[74] Syrgianidis GD, Mainou AC, Audergon JM (1993). Deux nouvelles varieties d’abricotier resistances à la maladie à virus de la sharka (Plum pox) issues de croissements. Deuxièmes rencontres sur l’abricotier, Avignon (France).

[75] Badenes ML, Martínez-Cavo J, García-Carbonell S, Villarrubia D, Llácer G (1997). Descripción de variedades autóctonas valencianas de albaricoquero.

[76] Della Strada G, Pennone F, Fideghelli C, Monastra F, Cobianchi D (1989). Monografía di cultivar di albicocco.

[77] Massai R (2010). Variability of apricot cultivars traits inside the ‘List of recommended fruit varieties’ project. Acta Hort (ISHS).

[78] Nyujtó F, Brozic S, Nyéki J, Brozic S (1982). Flowering and fruit set in apricot varieties grown in Hungary, and combination of varieties within the plantation. Acta Hort (ISHS).

[79] Alburquerque N, Egea J, Pérez-Tornero O, Burgos L (2002). Genotyping apricot cultivars for self-(in)compatibility by means of RNases associated with S alleles. Plant Breed.

