# Supplementary material for: Self-(in)compatibility in apricot germplasm is controlled by two major loci, S and M
Source: BMC Plant Biol. 2017 Apr 26;17:82. doi: 10.1186/s12870-017-1027-1 (PMC5405505; doi:10.1186/s12870-017-1027-1)
Supplement: Supplementary file 1 — Fragment sizes (bp) determined for S-RNase and SFB allele introns. Fragment sizes for the first S-RNase and the 5′-UTR SFB introns were exactly determined by an ABI PRISM 3100 Genetic Analyzer and those for the second S-RNase intron were approximately estimated from 0.8% agarose gels. References reporting S-allele molecular sizes for the first time are provided. (DOCX 15 kb) [file 12870_2017_1027_MOESM1_ESM.docx]

**Table S1** Fragment sizes (bp) determined for *S-RNase* and *SFB* allele introns.

| *S-allele* | *SRNase* intron 1 SR1-F/SR1-R | *SRNase* intron 1 PruT2/SR1-R | *SRNase* intron 2 PruC2/PruC4R^a^ | *SRNase* intron 2 PruC2/PruC6R^a^ | 5’UTR *FBox* intron | Refs.^b^ |
| --- | --- | --- | --- | --- | --- | --- |
| ***S*_1_** | 419 | 407 | ~2100 | ~2000 | 210 | [19] |
| ***S*_2_** | 345 | n.a. | ~950 | ~800 | 205 | [19] |
| ***S*_3_** | 285 | n.a. | n.a. | n.a. | 207 | [19] |
| ***S*_4_** | 260 | 244 | ~450 | ~300 | n.a. | [19] |
| ***S*_5_** | 396 | 384 | ~1300 | ~1200 | n.a. | [19] |
| ***S*_6_** | n.a.^c^ | 429 | ~1300 | ~1200 | 197 | [19] |
| ***S*_7_** | 419 | 407 | ~900 | ~700 | 199 | [19] |
| ***S*_C_** | 371 | 359 | ~3000 | ~2800 | 204 | [19] |
| ***S*_8_** | 371 | 359 | ~3000 | ~2800 | 204 | [28] |
| ***S*_9_** | 218 | 206 | ~600 | ~400 | n.a. | [28] |
| ***S*_11_** | 320 | 308 | n.a. | ~1500 | 205 | [28] |
| ***S*_15_/*S*_18_^d^** | 346/338 | 334/326 | ~750(*S*_15_)/400(*S*_18_) | ~550(*S*_15_)/200(*S*_18_) | 198/206 | [28] |
| ***S*_17_** | 427 | 417 | ~750 | ~550 | 189 | [28] |
| ***S*_19_** | n.a. | n.a. | n.a. | ~1800^e^ | n.a. | [28] |
| ***S*_20_** | n.a. | n.a. | ~1900 | ~1800 | n.a. | [41] |
| ***S*_22_** | 373 | n.a. | n.a. | ~500 | 206 | [*] |
| ***S*_24_** | n.a. | 266 | ~450 | ~300 | n.a. | [43] |
| ***S*_V_/*S*_X_^f^** | 260/n.a. | 248/n.a. | ~2000/550 | ~1800/350 | n.a./n.a. | [*] |

^a^ Fragment sizes for the second *S*-*RNase* intron were approximately estimated (~) from agarose gels. Thus size differences between the two PCR-amplified fragments (PruC2/Pruc4R and PruC2/PruC6R) for every *S*-allele range from 100 to 200 bp, though the exact difference is known to be of 150 bp according to the primer positions.

^b^ References reporting *S*-allele molecular sizes for the first time: Vilanova et al. [19]; Halázs et al. [28]; Zhang et al. [41]; Wu et al. [43]; This work [*].

^c^ n.a. Not amplified.

^d^ Only allele fragment sizes corresponding to the second intron could be unambiguously assigned to *S*_15_ and *S*_18_ according to Halázs et al. [28].

^e^ This fragment could only be weakly amplified.

^f^ Allele fragment sizes corresponding to *S*_V_ and *S*_X_ could not be established since both were found only once and in the same cultivar (‘Fergani’) (see Table 3).
