# Supplementary material for: Self-(in)compatibility in apricot germplasm is controlled by two major loci, S and M
Source: BMC Plant Biol. 2017 Apr 26;17:82. doi: 10.1186/s12870-017-1027-1 (PMC5405505; doi:10.1186/s12870-017-1027-1)
Supplement: Supplementary file 2 — Main results of homology searches performed by BLASTN against the NCBI Genbank database using Prunus armeniaca S-RNase sequences. Query sizes, Query cover %, Total score, E-value and Identity % are indicated. Putative names were assigned to the S-alleles according to the fragment sizes and sequence analyses. (DOCX 18 kb) [file 12870_2017_1027_MOESM2_ESM.docx]

**Table S2** Main results of homology searches performed by BLASTN against the NCBI Genbank database using *Prunus armeniaca* *S*-RNase sequences.

| **Query Sequence** | **Query size** | **Genbank Subject Sequence^c^** | **Total score** | **Query cover** | **E-value** | **Identity** | **Genbank Accession** |
| --- | --- | --- | --- | --- | --- | --- | --- |
| ***S*_17_^a^ (Orange red, Henderson & SEO, USA)**  This work (gDNA intron2) | 595 | ***S*_44_ (Shailaiyulvke, CH)**  *S*_17_ (Aurora, USA)  *S*_9_ (Xinshiji, CH) | 1072  1072  1055 | 98%  98%  98% | 0.0  0.0  0.0 | 99%  99%  99% | HQ342874.1  DQ270001.1  AY853594.1 |
| ***S*_31_^a^ (Cow-1 & Cow-2, FRA)**  This work (gDNA intron2) | 282 | ***S*_22_ (Tuxiangbai, CH)**  *S*_22b_ (Shajinhong, CH) | 490  490 | 98%  98% | 7e-135  7e-135 | 98%  98% | HM053569.1  GU574197.1 |
| ***S*_24_^a^ (Ezzine, TUN)**  This work (gDNA intron1) | 248 | ***S*_24_ (Chuanzhihong, CH)** | 453 | 100% | 8e-124 | 99% | HQ615602.1 |
| ***S*_20_^a,b^**  This work (gDNA intron2R) | 776 | ***S*_20_ (Shuixing, CH)**  *S*_55_ (Shushanggan, CH) | 1421  1399 | 100%  100% | 0.0  0.0 | 99%  99% | EF160078.1  KT223014.1 |
| ***S*_20_^a,b^**  This work (gDNA intron2F) | 893 | ***S*_20_ (Shuixing, CH)**  *S*_55_ (Shushanggan, CH) | 1626  1628 | 99%  99% | 0.0  0.0 | 99%  99% | EF160078.1  KT223014.1 |
| ***S*_1_ (Goldrich, USA)**  AY587561 (full CDS) | 712 | **SRNase-PA3 (HongYuxing,CH)**  SRNase-PADSI (Katyxing, CH) | 926  641 | 73%  51% | 0.0  5e-180 | 99%  99% | AF468676.1  AF468677.1 |
| ***S*_4_ (Harcot, CAN)**  AY587564 (full CDS) | 696 | ***S*_24_ (Chuanzhihong, CH)**  *S*_38_ (Guanyeliansihao, CH)  *S*_65_ (Saimaiti,CH)  *S*_48_ (Shacheheiyexing, CH)  *S*_24_ (Zhanggongyuan,CH) | 1288  927  763  757  757 | 100%  73%  61%  61%  60% | 0.0  3e-177  5e-180  2e-178  4e-176 | 99%  99%  99%  99%  99% | HQ615602.1  GU586228.1  JQ327151.1  HQ342878.1  EU037263.1 |
| ***S*_9_ (Ceglédi óriás, HUN)**  DQ 269996 (gDNA intron2) | 498 | ***S*_14_ (Baixing, CH)**  *S*_43_ (Jiagedamayishang, CH)  *S*_14a_ (Kezikeximixi, CH)  *S*_66_ (Xiaobiaxing, CH) | 870  870  861  837 | 99%  97%  97%  94% | 0.0  0.0  0.0  0.0 | 99%  99%  99%  99% | DQ870630.1  JQ327152.1  GU574199.1  HQ342873.1 |
| ***S*_12_ (Korai zamatos, HUN)**  DQ269998 (gDNA intron2) | 360 | ***S*_24_ (Chuanzhihong, CH)**  *S*_38_ (Guanyeliansihao, CH)  *S*_42_ (Maolaxiao, CH)  *S*_24_ (Zhanggongyuan,CH)  *S*_4_ (Harcot, CAN)  *S*_65_ (Saimaiti,CH)  *S*_48_ (Shacheheiyexing, CH) | 636  636  634  603  599  590  590 | 97%  97%  97%  93%  97%  92%  92% | 1e-178  1e-178  4e-178  1e-168  9e-167  9e-165  9e-165 | 99%  99%  99%  99%  97%  99%  99% | HQ615602.1  GU586228.1  HQ342872.1  EU037263.1  AY587564.1  JQ327151.1  HQ342878.1 |
| ***S*_16_ (Zard, HUN)**  EF173401 (partial CDS) | 563 | ***S*_54_ (Shushanggan, CH)** | 731 | 71% | 2e-167 | 99% | KT223013.1 |
| ***S*_17_ (Aurora, USA)**  DQ270001 (gDNA intron2) | 659 | ***S*_44_ (Shailaiyulvke, CH)**  *S*_9_ (Xinshiji, CH) | 1194  1188 | 98%  99% | 0.0  0.0 | 99%  99% | HQ342874.1  AY853594.1 |
| ***S*_20_ (Ceglédi Piroska, HUN)**  EF173402 (gDNA) | 764 | ***S*_36_ (Yinxiangbai, CH)**  *S*_17_ (Yingxiangbaixing, CH) | 1016  883 | 74%  63% | 0.0  0.0 | 99%  99% | GU574198.1  DQ870633.1 |
| **S_5_ (Colorao, SPA)**  Vilanova et al. [17]  (gDNA intron1) | 472 | ***S*_13_ (Korai zamatos, HUN)** | 334 | 39% | 3e-61 | 100% | EF173339.1 |

^a^ Putative name assigned to the *S*-alleles according to the fragment sizes and sequence analyses.

^b^ Cultivars and accessions from which *S*_20_ fragments were sequenced include: ‘Cristalí’, ‘Gavatxet’, ‘Tadeo’ and ‘Velázquez’ (Spain); ‘Harlayne’ and ‘Veecot’ (Canada); ‘Stella’ (USA); ‘Perla’ and ‘Portici’ (Italy); ‘Mariem’ (Tunisia) and ‘Cow-2’ (France).

^c^ All showed matches correspond to *Prunus armeniaca* *S*-RNases.
