# Supplementary material for: Self-(in)compatibility in apricot germplasm is controlled by two major loci, S and M
Source: BMC Plant Biol. 2017 Apr 26;17:82. doi: 10.1186/s12870-017-1027-1 (PMC5405505; doi:10.1186/s12870-017-1027-1)
Supplement: Supplementary file 3 — Characteristics of SSR primers developed from peach (PGS) and apricot genome sequences (AGS) located at the M-locus. Repeat motifs, Primer sequences, Scaffold positions, ORF (Prupe), Size range, N° of alleles and Heterozygosity (in the set of accessions analyzed) are indicated. (DOCX 14 kb) [file 12870_2017_1027_MOESM3_ESM.docx]

**Table S3** Characteristics of SSR primers developed from peach (PGS) and apricot genome sequences (AGS) located at the *M*-locus

| Name | F/R | Primer sequence | Repeat motif | Start on scaffold_3 (Mb)^3^ | ORF (Prupe) | Size range (bp) | No. of alleles | *H*^4^ |
| --- | --- | --- | --- | --- | --- | --- | --- | --- |
| PGS3.22^1^ | F | TCTGATTGCAGGTAAGGACAG | (CT)_25_ | 18.49 | 3G247600 | 304-328 | 8 | 0.61 |
|  | R | TATCTTGATATCGGCCTGGA |  |  | Put. Prot. |  |  |  |
| PGS3.23^1^ | F | TGACTTTCTGCATCTTGACCT | (AG)_24_ | 18.61 | 3G249300 | 164-190 | 8 | 0.68 |
|  | R | CTTTGCTTCCGTTAATCCAA |  |  | MADS-box |  |  |  |
| PGS3.62^1^ | F | AGCTTCCTCTATTCTTGGTGGT | (CT)_22_ | 18.61 | ---- | 321-356 | 10 | 0.71 |
|  | R | GCTTTTCCCCGAGCTAATTC |  |  |  |  |  |  |
| PGS3.71^1^ | F | ACCACCCCCTATCCCTATTG | (CT)_13_ | 18.40 | ---- | 233-269 | 10 | 0,65 |
|  | R | ACTTGCAAACCCCCTTGATT |  |  |  |  |  |  |
| PGS3.96^1^ | F | TGGCCACAATTAATGGGAGA | (CT)_14_ | 18.76 | ---- | 431-474 | 15 | 0,82 |
|  | R | TCGGAGAACTTCTTGTGCAT |  |  |  |  |  |  |
| AGS3.20^2^ | F | CGAACGAGAGGGAAAAATGA | (TA)_10_ | 18.61 | 3G249300 | 178-202 | 8 | 0.62 |
|  | R | AACTGATTCCGAACCACAGG |  |  | MADS-box |  |  |  |
| AGS3.30^2^ | F | CCGCACGGCTATACTGTCTAA | (AT)_13_ | 18.71 | ---- | 193-207 | 7 | 0.38 |
|  | R | ACAGGCTGGATGCTTTGTCT |  |  |  |  |  |  |

^1^ Zuriaga et al. [26]

^2^ This work

^3^ Positions according to the peach v1.0 genome sequence (IPGI)

^4^ Heterozygosity was estimated according to Nei [46]: *H = 1-Σp_i_^2^*, where *p_i_* is the frequency of the *i^th^* allele. Clonal sibs from ‘Canino’ were excluded from estimations.
