# Supplementary material for: Self-(in)compatibility in apricot germplasm is controlled by two major loci, S and M
Source: BMC Plant Biol. 2017 Apr 26;17:82. doi: 10.1186/s12870-017-1027-1 (PMC5405505; doi:10.1186/s12870-017-1027-1)
Supplement: Supplementary file 4 — Allele sizes (bp) for the SSR markers comprised into the M-haplotypes. ‘Main class’ as well as the subtype are indicated for every M-haplotype. Absolute frequency in the set of accessions analyzed is also provided. (DOCX 18 kb) [file 12870_2017_1027_MOESM4_ESM.docx]

**Table S4** Allele sizes (bp) for the SSR markers comprised into the *M*-haplotypes

| Main class | M-haplotype | PGS3.71 | PGS3.22 | PGS3.62 | PGS3.23 | AGS.20 | AGS.30 | PGS3.96 | Abs. freq.^a^ |
| --- | --- | --- | --- | --- | --- | --- | --- | --- | --- |
| *m*0 | *m*0-0 | 261 | 306 | 348 | 188 | 190 | 203 | 442 | 26 |
|  | *m*0-1 | 261 | 306 | 348 | 188 | 190 | 203 | 444 | 1 |
| *M*1 | *M*1-0 | 259 | 306 | 350 | 188 | 188 | 203 | 441 | 12 |
|  | *M*1-1 | 257 | 306 | 348 | 188 | 188 | 203 | 441 | 2 |
|  | *M*1-2 | 259 | 306 | 348 | 188 | 188 | 203 | 441 | 1 |
|  | *M*1-3 | 259 | 306 | 348 | 178 | 188 | 203 | 441 | 1 |
|  | *M*1-4 | 257 | 306 | 348 | 184 | 188 | 203 | 441 | 1 |
| *M*2 | *M*2-0 | 255 | 310 | 336 | 178 | 188 | 203 | 434 | 7 |
|  | *M*2-1 | 255 | 310 | 336 | 178 | 188 | 201 | 434 | 1 |
|  | *M*2-2 | 255 | 310 | 336 | 178 | 188 | 203 | 436 | 1 |
| *M*3 | *M*3 | 247 | 312 | 329 | 190 | 192 | 195 | 458 | 8 |
| *M*4 | *M*4-0 | 259 | 306 | 336 | 178 | 188 | 195 | 456 | 10 |
|  | *M*4-1 | 259 | 306 | 336 | 178 | 188 | 195 | 458 | 5 |
|  | *M*4-2 | 259 | 306 | 336 | 178 | 188 | 195 | 460 | 1 |
| *M*5 | *M*5-0 | 233 | 310 | 354 | 184 | 190 | 203 | 466 | 5 |
|  | *M*5-1 | 233 | 310 | 356 | 184 | 192 | n.a. | 466 | 2 |
|  | *M*5-2 | 233 | 310 | 354 | 184 | 190 | n.a. | 464 | 2 |
| *M*6 | *M*6 | 269 | 304 | 321 | 164 | 202 | 203 | 443 | 2 |
| *M*7 | *M*7-0 | 251 | 316 | 332 | 186 | 198 | 193 | 464 | 2 |
|  | *M*7-1 | 233 | 316 | 332 | 186 | 198 | 193 | 464 | 1 |
|  | *M*7-2 | 233 | 316 | 332 | 186 | 196 | 193 | 464 | 1 |
|  | *M*7-3 | 251 | 316 | 332 | 186 | 194 | 195 | 466 | 1 |
| *M*8 | *M*8-0 | 259 | 328 | 354 | 186 | 198 | 195 | 431 | 4 |
|  | *M*8-1 | 259 | 328 | 354 | 186 | 196 | 195 | 431 | 2 |
|  | *M*8-2 | 259 | 328 | 356 | 186 | 196 | 195 | 431 | 1 |
| *M*9 | *M*9 | 261 | 312 | 356 | 184 | 196 | n.a. | 466 | 3 |
| *M*10 | *M*10 | 247 | 312 | 354 | 178 | 188 | 203 | 474 | 4 |
| *M*11 | *M*11 | 233 | 314 | 332 | 190 | 188 | 199 | 474 | 1 |
| *M*12 | *M*12 | 259 | 312 | 356 | 184 | 194 | 203 | 468 | 6 |
| *M*13 | *M*13 | 255 | 310 | 348 | 188 | 188 | 203 | 441 | 2 |
| *M*14 | *M*14-0 | 255 | 306 | 348 | 188 | 188 | 195 | 466 | 1 |
|  | *M*14-1 | 255 | 306 | 348 | 188 | 188 | 195 | 450 | 1 |
| *M*15 | *M*15-0 | 259 | 306 | 356 | 186 | 192 | 203 | 466 | 1 |
|  | *M*15-1 | 257 | 306 | 354 | 186 | 192 | 203 | 466 | 1 |
| *M*16 | *M*16 | 243 | 310 | 337 | 180 | 188 | 195 | 442 | 1 |
| *M*17 | *M*17 | 256 | 308 | 348 | 168 | 178 | 203 | 434 | 1 |
| *M*18 | *M*18 | 257 | 310 | 354 | 190 | 188 | 207 | 468 | 1 |
| *M*19 | *M*19 | 255 | 306 | 334 | 188 | 190 | n.a. | n.a. | 1 |

^a^ Absolute frequency for every *M*-haplotype in the set of accessions analyzed excluding clonal sibs from ‘Canino’ (64). Four out of the total 128 *M*-haplotypes, corresponding to the cultivars ‘Kech-pshar’ and ‘Fergani’, could not be determined.
