# Supplementary material for: Self-(in)compatibility in apricot germplasm is controlled by two major loci, S and M
Source: BMC Plant Biol. 2017 Apr 26;17:82. doi: 10.1186/s12870-017-1027-1 (PMC5405505; doi:10.1186/s12870-017-1027-1)
Supplement: Supplementary file 5 — Primers used in this study. Sequences and references are indicated. (DOCX 14 kb) [file 12870_2017_1027_MOESM5_ESM.docx]

**Table S5** Primers used in this study

| Region | Primer | Sequence | Reference |
| --- | --- | --- | --- |
| S-RNase 1^st^ exon | SRc-F | 5´-CTC GCT TTC CTT GTT CTT GC -3´ | Romero et al. [66] |
| S-RNase 2^nd^ exon | SRc-R | 5´-GGC CAT TGT TGC ACA AAT TG -3´ | Vilanova et al. [19] |
| S-RNase 1^st^ exon | Pru-T2 | 5´-GTT CTT GCT TTT GCT TTC TTC-3´ | Tao et al. [67] |
| S-RNase 2^nd^ exon | Pru-C2 | 5´-CTT TGG CCA AGT AAT TAT TCA AAC C-3´ | Tao et al. [67] |
| S-RNase 2^nd^ exon | Pru-C2R | 5´-GGT TTG AAT AAT TAC TTG GCC ATA G-3´ | Tao et al. [67] |
| S-RNase 3^rd^ exon | Pru-C4R | 5´-GGA TGT GGT ACG ATT GAA GCG-3 | Tao et al. [67] |
| S-RNase 3^rd^ exon | Pru-C6R | 5´-CAT TGC CAC TTT CCA CGT C-3´ | Vilanova et al. [19] |
| 5´UTR-Fbox intron | F-BOX5´A | 5´-TTK SCH ATT RYC AAC CKC AAA AG -3´ | Vaughan et al. [70] |
| SFB exon | F-BOXintronR | 5´-CWG GTA GTC TTD SYA GGA TG- 3´ | Vaughan et al. [70] |
| SFB_C_ exon | RFBc-F | 5´-GAG GAG TGC TAC AAA CTA AGC-3´ | Vilanova et al. [17] |
| SFB_C_ exon | SFBins-R | 5´-TCA AGA ACT TGG TTG GAT TCG-3´ | Vilanova et al. [17] |
| 5’-UTR S_2_-RNase | Sf-Hap2 | 5´-CGC TAG AAA TCA AAG CCA CAG-3´ | Vilanova et al. [17] |
| 3’-UTR S_2_-RNase | Sr-Hap2 | 5´-GGC GTA AGC AAG TGG AAA AG-3´ | Vilanova et al. [17] |
| 5’-UTR SFB_2_ | FBf-Hap2 | 5´-GCC CAA TTA CTT GGT CAC TG-3´ | Vilanova et al. [17] |
| 3’-UTR SFB_2_ | FBr-Hap2 | 5´-CAC CCA CTT GAC TTG TCA GC-3´ | Vilanova et al. [17] |
| SFB exon | SFBc-F | 5´-TCG ACA TCC TAG TAA GAC TAC CTG C-3´ | Romero et al. [66] |
| SFB_2_ exon | FBF5 | 5´-TAG GAC CCC TCA AAT GAG C-3´ | Unpublished |
| SFB_2_ exon | FBF6 | 5´-TGG GTT CTG CAA GAA AAA CG-3´ | Unpublished |
